# The Mechanism of Lipopolysaccharide Escaping the Intestinal Barrier in *Megalobrama amblycephala* Fed a High-Fat Diet

**DOI:** 10.3389/fnut.2022.853409

**Published:** 2022-04-07

**Authors:** Yong-Jun Dai, Wen-Bin Liu, Kenneth Prudence Abasubong, Ding-Dong Zhang, Xiang-Fei Li, Kang Xiao, Xi Wang, Guang-Zhen Jiang

**Affiliations:** Key Laboratory of Aquatic Nutrition and Feed Science of Jiangsu Province, National Experimental Teaching Center for Animal Science, College of Animal Science and Technology, Nanjing Agricultural University, Nanjing, China

**Keywords:** high-fat diet, lipopolysaccharide, chronic inflammation, mucus barrier, transcellular pathway

## Abstract

With the popularity of western food characterized by excessive fat and sugars, obesity has currently been a public health issue. Low-grade chronic inflammation accompanied by obesity increases the risk of multiple epidemics such as diabetes, cancer and cardiovascular diseases. Here, we show that feeding *Megalobrama amblycephala* with a high-fat diet (HFD) drives obesity-related chronic inflammation and the penetration of lipopolysaccharide (LPS). Interference with antibiotics inhibits the produce of LPS and this alleviates the sustained release of pro-inflammatory factors induced by HFD. LPS penetration is attributed to weakened intestinal mucus barrier after high-fat exposure. Mechanically, the consumption of HFD inhibits the secretion of mucin 2 (MUC2) due to the induction of endoplasmic reticulum stress mediated by the inositol-requiring enzyme 1 (IRE1) /X box-binding protein 1 (XBP1) pathway in goblet cells. Furthermore, excessive lipid exacerbates the leakage of LPS across the intestinal epithelial cell barrier via the transcellular pathway. Mechanically, lipid increases the internalization of LPS in intestinal epithelial cells depending on the activation of fatty acid translocase (FAT/CD36). These results demonstrate that HFD causes the penetration of LPS due to the weakened intestinal mucosal barrier and the assistance of CD36.

## Introduction

With the increased intake of excessive fat and sugars, obesity has been a public health issue as over 650 million populations are obese and is currently ranked as the fifth major risk factor for increased mortality globally (1). Obesity is characterized by a low-grade inflammation, which increases the risk for an extensive array of diseases including type 2 diabetes (T2D), cardiovascular disease (CVD), fatty liver disease and cancer (1). A large number of studies have confirmed that low-grade chronic inflammation is involved in the development of multiple pathologies accompanied with obesity, such as insulin resistance (2), endoplasmic reticulum (ER) stress (3), leptin resistance (4), cell death (5) and fibrosis (6). Inflammation is considered to be the key to solving obesity-related syndromes. The initial inducement of chronic inflammation is still under discussion, and no unanimous point of view is formed. It is reported that nutritional intervention alleviates the low-grade chronic inflammation by disturbing the intestinal microbes, especially gram-negative bacteria (7–9). Lipopolysaccharide (LPS), a product sourced from intestinal microbes and characterized as a potent mediator for inflammation, infiltrates into the lymphatic circulation after high-fat diets (HFD) is consumed (10). Here, we assume that penetration of LPS into the lymphatic circulation is responsible for inducing inflammation during high-fat feeding. Therefore, the purpose of this experiment is to understand the mechanism by which LPS penetrates into the lymphatic circulation.

LPS, also known as endotoxin, is a structural component present in the outer membrane of gram-negative bacteria (11). LPS constitutes of lipid A, core sugars and the O-antigen. Lipid A shares a similar structure with long-chain fatty acids, and is mainly responsible for LPS bioactivity including stimulating the release of pro-inflammatory factors (12). LPS at low concentrations can induce inflammation and even cause sepsis and multiple organ dysfunction (13). Under normal conditions, LPS is fenced by the intestinal barrier and therefore exists in the gut lumen where most bacteria hosts. Once intestinal permeability was increased due to damage of the intestinal barrier, LPS translocated to blood circulation and then induced pathology associated with inflammation. In the mucosal layer, the translocation of LPS is prevented by mucus composed mainly of MUC2 (14). In addition, LPS is also intercepted by the defense formed by intestinal epithelial cells (IECs) binding closely (15). Most exogenous macromolecules cannot be absorbed by cells and therefore cross the intestinal epithelial barrier via the paracellular space (16). “Non-canonical inflammasome” was reported that LPS directly interact with cytoplasmic caspase 11 to activate intracellular pathways independent on an extracellular receptor, which hint metastasis of LPS into the cytosol and a novel mechanism facilitating LPS to escape the intestinal barrier via transcellular pathway (17). Since “Non-canonical inflammasome” was found, numerous researches focused on the signal transmission of intracellular LPS. However, the mechanism by which LPS transfer across the cell membrane and migrate into the cytoplasm is still unknown. Understanding the mechanism of LPS cellular internalization will provide us with a novel perspective for LPS escaping the intestinal epithelial cell barrier. This possibly provide new strategies to alleviate metabolic endotoxemia.

Chronic inflammation accompanied by obesity has posed a threat to diverse diseases. In this experiment, we investigate this question using blunt snout bream (*Megalobrama amblycephala*). *M. amblycephala* is a common fish which belongs to the *Cyprinidae* family. Our previous study reported that HFD (10% fat) induced typical obesity-related symptoms in *M. amblycephala*, as found in other high-fat models (18–20). Therefore, it provides a suitable whole-body system for investigating mechanisms of HFD-induced inflammation.

## Materials and Methods

### *In vivo* Experiments

The care and use of animals as well as all experimental procedures involving animals has been approved by the Animal Care and Use Committee of Nanjing Agricultural University (Nanjing, China).

High-fat model establishment: To establish a high-fat model, experimental diets with normal-fat (5% fat, NFD) or high-fat (10% fat, HFD) were manufactured. Formulation and proximate composition of the experimental diets were shown in Table 1. Juvenile *Megalobrama amblycephala* were obtained from a local fish hatchery (Nanjing, China). After a 20 days acclimation, fish of similar sizes (average initial weight: 20.0 ± 0.21 g) were randomly distributed into cages which were anchored in an outdoor pond. The experiment was divided into two groups with 4 replicates (*N* = 4). Fish in control group were fed with NFD, while those in the treatment group were fed with HFD. All fish were hand-fed to apparent satiation three times daily (08:00, 12:00 and 17:00) for 12 weeks. Feeding rates about 5% body weight was fed per day. Water temperature varied from 27 to 31°C, and pH fluctuated between 7.2 and 7.6. Ammonia nitrogen and nitrite nitrogen were maintained below 0.4 mg/L and 0.064 mg/L, respectively. Dissolved oxygen was maintained above 6.00 mg/L during the feeding trial.

**Table 1:** Formulation and proximate composition of the experimental diets.

**Ingredients (%)**	**High-fat diet (%)**	**Normal-fat diet (%)**
Fish meal	5.00	5.00
Soybean meal	30.00	30.00
Rapeseed meal	15.00	15.00
Cottonseed meal	10.60	8.12
Soybean oil	9.48	4.34
Wheat bran	3.98	7.65
Wheat flour	22.04	26.00
Ca(H_2_PO_4_)_2_	1.80	1.80
Salt	0.40	0.40
Ethoxyquinoline	0.50	0.50
Premix^a^	1.20	1.20
Proximate composition		
Moisture	11.45	11.02
Crude protein	30.15	30.38
Crude lipid	10.46	5.29
Energy (MJ/kg)	20.01	18.15

a *Premix supplied the following minerals (g/kg) and vitamins (IU or mg/kg): CuSO_4_·5 H_2_O, 0.02 g; FeSO_4_·7 H_2_O, 0.25 g; ZnSO_4_·7 H_2_O, 0.22 g; MnSO_4_·4 H_2_O, 0.07 g; Na_2_SeO_3_, 0.0004 g; KI, 0.00026 g; CoCl_2_·6 H_2_O, 0.001 g; Vitamin A, 900000IU; Vitamin D, 200000IU; Vitamin E, 4,500 mg; Vitamin K3, 220 mg; Vitamin B_1_, 320 mg; Vitamin B_2_, 1090 mg; Niacin, 2,800 mg; Vitamin B_5_, 2000 mg; Vitamin B_6_, 500 mg; Vitamin B_12_, 1.6 mg; Vitamin C, 5,000 mg; Pantothenate, 1,000 mg; Folic acid, 165 mg; Choline, 60,000 mg*.

Antibiotic intervention: Antibiotics were used to inhibit the number of bacteria, especially gram-negative bacteria. Normal-fat diets (5% fat), high-fat diet (10% fat) and high-fat diet (10% fat) supplemented with antibiotics (vancomycin 2 g/kg, erythromycin 4 g/kg, neomycin sulfate 4 g/kg and metronidazole 4 g/kg, HFDA) were manufactured and fed to experimental fish. The experiment was divided into three groups with 4 replicates (*N* = 4). Experimental fish with an initial weight of 27 ± 0.58 g were used. Management of experimental fish was the same with the experiment of high-fat model.

Induction of intestinal injury: Dextran Sulfate Sodium (DSS) was used to induce intestinal injury. All fish (average weight, 23.74 ± 0.76 g) were divided into two groups with 4 replicates (*N* = 4) and cultured in circular fiberglass tanks (300 L water/tank). The experimental fish were cultured in water dissolved with DSS at a dose of 0.5% (w/v) for 14 days. One-third of the water was refreshed every day to avoid decomposition-associated variation in DSS solution quality.

The oral administration of fluorescein isothiocyanate isomer- dextran (FITC-d): At the end of the experiment, fish were fasted for 24 h to empty the digestive tract. Four fish (*N* = 4) per group were gavaged with 900 mg/kg FICT-d for 4 h. After fish were anesthetized in diluted tricaine methanesulfonate (MS-222), the blood was collected by caudal venipuncture and centrifuged at 2,000 g, 4°C for 10 min. The concentration of FICT-d in the plasma was determined using a fluorescence microplate reader and a standard curve from a serial dilution of FICT-d in PBS.

The oral administration of LPS: At the end of the experiment, fish were fasted for 24 h to empty the digestive tract. Four fish (*N* = 4) per group were gavaged with 1 mg/kg LPS or FICT-LPS for 4 h. The blood was collected by caudal venipuncture and centrifuged at 2,000 g, 4°C for 10 min. The concentration of LPS was determined by enzyme-linked immunosorbent assay (ELISA). The concentration of FICT-LPS was determined using a fluorescence microplate reader and was calculated by relative fluorescence value. The intestine was collected at low temperature and then frozen directly at optimal cutting temperature (OCT). Samples embedded in the OCT were sliced into 8 μm sections at −20°C. FICT—LPS was observed with a laser confocal microscope.

### Culture and Treatment

Cell lines were cultured in Dulbecco's Modified Eagle Medium (DMEM, GIBCO) containing 10% FBS and 1% antibiotics (penicillin and streptomycin) at 37°C in 5% CO_2_. DMEM medium without phenol red was used because the color of the medium will interfere with the determination of the medium index. All cell experiments were performed with four replicates. The non-toxic concentration range of LPS and LA was determined by incubation by the gradient. Cells were cultured in 96-well plates and then LPS (0, 10, 20, 40, 80, and 160 ng/ml) or oleic acid (OA) (0, 25, 50, 100, 200, and 400 mmol/L) was administrated for 24. To observe the mechanism of LPS entering the cytosol, cells were transfected with 40 ng FICT-LPS or 100 mmol/L LA for 24. After incubation, the culture medium was discarded, the excess FICT-LPS was washed with PBS and then inspected by a laser confocal microscope. After incubation, cells were digested with 0.25 trypsin, then resuspended in PBS and then analyzed by Flow cytometer. To verify the assistance of CD36 on the entry of LPS into the cytosol, cells were transfected with siRNA CD36, FICT-LPS and LA for 24 h. After incubation, FICT-LPS, cell viability and cytotoxicity were detected. In order to detect the release of IL-1β, the medium without phenol red was used.

### Histopathology and Immunohistochemistry

After fish were euthanized by decapitation, the intestine was immediately dissected, fixed in a 10% paraformaldehyde solution (12 h), washed in PBS buffer (0.1 M, pH 7.2) and dehydrated with a 70% ethanol gradient and embedded in paraffin (Paraplast/McCormick) The samples that were embedded in paraffin were sectioned to a thickness of 5 μm. The sections were stained with haematoxylin for 5 min, washed with PBS and then stained with eosin at room temperature (3 sections per fish); In order to observe intensity of goblet cells, the sections were stained with periodic-acid schiff (PAS) (3 sections per fish).

For immunohistochemistry, the samples that were embedded in paraffin were sectioned to a thickness of 5 μm. After deparaffinization and hydration, the sections were pre-treated with 3% H_2_O_2_ in methanol at room temperature for 10 min and then heated in 10 mmol/L citrate buffer (pH 6.0). After several rinses in PBS, the sections were blocked with 10% goat serum at room temperature for 20 mins, and then incubated overnight at 4°C primary antibody (MUC2, No. ab272692, Abcam; GPR 78, No.11587-1-AP, Proteintech; occludin, No. 27260, Proteintech). After rinsing with PBS several times, the sections were incubated with a biotinylated secondary antibody at 37°C for 30 min. After rinsing several times in PBS, immunodetection was conducted.

### Western Blotting

The protein expressions were determined using western blot (WB), 100 mg tissue samples or 6-well plate cells were washed and then homogenated with RIPA lysis buffer. The homogenate was centrifuged at 12,000 × g for 30 min, and then the supernatant was retained. Protein concentrations were determined using a BCA protein assay kit (Beyotime Biotechnology, China). The target proteins were combined with the primary antibody: occludin (No. 27260, Proteintech), GRP 78 (No.11587-1-AP, Proteintech), IRE1 (No. DF7709, Affinity), phosphor-IRE1 (No. AF7150, Affinity), MUC2 (No. ab272692, Abcam) β-actin (No. 66009, Proteintech) and then labeled by diluted goat anti-rabbit IgG. The intensity of target bands were quantified using the Image J 1.44p software (U.S. National Institutes of Health, Bethesda, MD, USA).

### Quantitative PCR Analyses

DNA in intestinal was extracted using reagent test kit (Tiangen, No. DP328-02) following its instructions. Total RNA was extracted from hepatopancreas using Trizol reagent (Invitrogen, California, USA) following its instructions. The quantity of isolated RNA was determined by absorbance measures at 260 and 280 nm. RNA was reversely transcribed using a PrimeScript^TM^ RT reagent Kit (Takara, Japan) following its instructions. The polymerase chain reaction was determined using an SYBR Green II Fluorescence Kit (Takara Bio. Icn, Japan). Primers were designed based on the available sequences as follows. Total bacteria: GTGSTGCAYGGYYGTCGTCA, ACGTCRTCCMCNCCTTCCTC; TNF-α: TCAAAGTCAGGCGTATGG, CTGGCTGTAGACGAAGTAAAT; IL-6: ATGACGGCGTATGAAGG, AGAGGACCGCTGGAGAT; EF1α: CTTCTCAGGCTGACTGTGC, CCGCTAGCATTACCCTCC. The mRNA expression was normalized to EF1α using the 2^−ΔΔ^ CT method.

### Cell Viability and Cytotoxicity Assay

To detect cell viability, cells were cultured in 96-well plates. After cells were treatment with LPS for 24 h, cells were incubated with CCK8 (10%) for 2 h. The cell viability was detected using a Microplate reader at OD 450 nm.

To detect cell toxicity, cells were cultured in 96-well plates. Cell toxicity was evaluated by detecting the release of lactate dehydrogenase (LDH). Cell toxicity was calculated by the release of LDH after treatment /total LDH in the cell. First, total LDH in the cell was measured. The cells were collected after being cultured for 48 h, and then the cell membrane was destroyed to release total LDH. Secondly, cells were cultured in 96-well plates for 24 h, and then 40 ng/ml LPS was incubated for 24. The culture medium was collected and the released LDH was tested. The assay measured LDH via the conversion of lactate to NADH and then pyruvate NADH reacted with INT (2-p-iodophenyl-3-nitrophenyl tetrazolium chloride) to generate formazan, which was detected using a Microplate reader at OD 490 nm.

### Flow Cytometry Analysis

Goblet cells were sorted by using the BD FACSAria II sorter (BD Biosciences). Intestinal was separated, digested by 0.25% trypsin for 10 min, filtered through a nylon cell strainer (200 meshes), washed three times with PBS, and then incubated with MUC2 for 2 h to sort goblet cells.

Cells were cultured in 6-well plates and then 40 ng/ml FITC-conjugated LPS (No. F3665, Sigma) or linoleic acid was incubated for 24 h. The medium was discarded, the excess FICT-LPS was washed with PBS, and then cells transfected with FITC-LPS were collected and analyzed in a flow cytometer.

Cell apoptosis was measured by Annexin V-FITC/PI apoptosis detection kit (No. A211-01, Vazyme Biotech, china). Cells were seeded in six-well plate for one day and then were incubated with LPS, linoleic acid or SSO for 24 h. Cells (2.5 × 105 cells/well) were collected and cleaned with PBS, and then the cells were suspended in Annexin V binding buffer (100 μL) and stained with PI (5 μL) and Annexin V-FITC (5 μL). After the cells were incubated for 20 min in dark, 400 ul Annexin V binding buffer was added for the subsequent flow cytometric analysis.

### Analysis of Pro-inflammatory Factors

Intestine and liver samples were weighed and homogenized (dilution 1:10) in 10 volumes (v/w) of in a tissue homogenizer and centrifuged at 3,000 × g at 4°C for 10 min. The supernatant was collected and then reserved immediately for analysis of pro-inflammatory factors. The content of pro-inflammatory factors was standardized by protein content. The concentrations of TNF-α, IL-6, IL-1β and MCP-1 were measured using cytokine-specific an enzyme-linked immunosorbent assay (ELISA) kits according to the manufacturer's instructions (R and D Systems, USA, no. MTA00B, D6050, DY401 and DCP00), after testing the linear functional relation between concentration and absorbance. The protein content was tested using BCA Protein Assay Kit (Beyotime Biotechnology, China, no. P0010).

### Statistical Analysis

Statistical analysis was conducted using the SPSS program version 22.0 (SPSS Inc.). Differences between two groups were analyzed using two-tailed Student's *t*-test. Differences among groups (more than 2 groups) were analyzed using one-way ANOVA followed by Tukey's *post hoc* test. Differences *P* < 0.05 was considered significant. Data are expressed as mean ± S.E.M. (standard error of mean).

## Results

### High-Fat Diet Induces Obesity Associated With Systematic Chronic Inflammation and LPS Penetration

**Figure 1:**
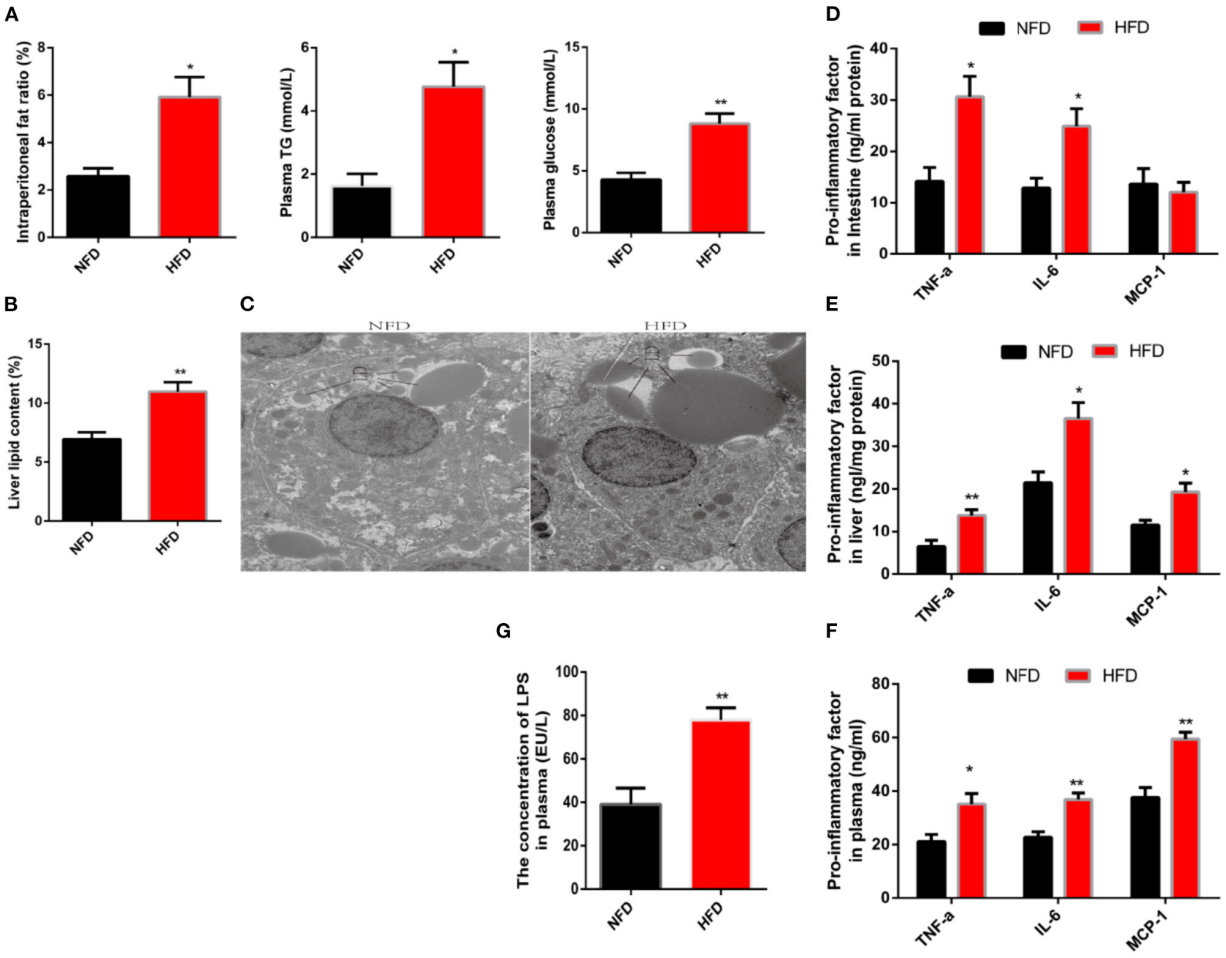
Long-term consumption of a high-fat diet induces characteristics of obesity and low-grade systemic inflammation. Fish were fed a normal-fat diet (5% fat, NFD) or high-fat diet (10% fat, HFD) for 12 weeks. **(A)** Intraperitoneal fat ratio, plasma glucosep and plasma triglycerides (TG). **(B)** The content of liver fat. **(C)** Analysis of lipid droplets by transmission electron microscope. **(D–F)** The protein content of pro-inflammatory factors (TNF-α, IL-6 and MCP-1) in the intestine **(D)**, plasma **(E)** and liver **(F)**. **(G)** Concentration of LPS in plasma. Data are shown as mean ± SEM. *N* = 4. Two-tailed unpaired Student's *t*-test was used. *P < 0.05, **P < 0.01.

To investigate the impact of high-fat diets (HFD) on low-grade chronic inflammation, we first established a high-fat model where the subjects were fed diets supplemented with 10 or 5% fat for 12 weeks. Compared with the normal fat diet (NFD, 5% fat) group, consumption of the HFD significantly increased the abdominal fat rate (Figure 1A). Hyperglycemia and hyperlipidemia are also typical physiological indicators associated with obesity (21). An agreement symptom was presented in this high-fat model, which was evidenced by a significant increase in plasma concentrations of glucose and triglyceride (Figure 1A). In addition, the unwanted accumulation of fat in tissues is also considered an important pathological step in the formation of obesity (22). Here, we used liver tissue as an example to observe fat accumulation. After lipid was separated, extracted and then the liver lipid was calculated, a significant increase in liver lipid was observed because of consumption HFD (Figure 1B). Further, we observed lipid droplets in liver cells with the transmission electron microscope. We found that the lipid droplets size in the HFD group were significantly larger than that in the NFD group (Figure 1C). Next, we explored the impact of HFD on obesity-related chronic inflammation. Tumor necrosis factor-α (TNF-α) and interleukin-6 (IL-6) were regarded as pro-inflammatory factors and were introduced as an indicators to evaluate inflammation (23). These two factors were detected by using an ELISA. Compared with the control group, the protein content of pro-inflammatory factors in the liver and intestine increased significantly (Figures 1D–F). In addition, consumption of HFD substantially increased the release of pro-inflammatory factors in the plasma, which implied a complication of systemic chronic inflammation. Increased release of pro-inflammatory factors would initiate the immune response by recruiting immune cells to target organs. This process is mediated by macrophage chemoattractant protein-1 (MCP-1) (24). Chronic consumption of HFD significantly increased the protein content of MCP-1 in plasma and liver, consistent with chronic inflammation (Figures 1E,F). Inflammation, as part of host defense mechanisms, guaranteed restoration of tissue homeostasis when the injury was caused by a harmful stimulus (25). If the inflammation cannot be terminated on time, the chronically increased release of pro-inflammatory factors excessively activates the immune response and eventually causes further disruption to homeostasis (26). Therefore, chronic inflammation may be a potential target to solve the sub-health caused by obesity. However, the inducement of chronic inflammation is still under discussion. LPS, also known as endotoxin, is the main component of outside membrane in gram-negative bacteria. Once LPS enters the internal environment, inflammation and even organ failure are induced. It is reported that the consumption of HFD increases the concentration of LPS in blood circulation (27). In this study, we also observed consistent results that the LPS penetrates into the blood increased in animals eating HFD (Figure 1G).

### The Penetration of LPS Is Responsible for the Chronic Inflammation of the System Induced by High-Fat Diets

**Figure 2:**
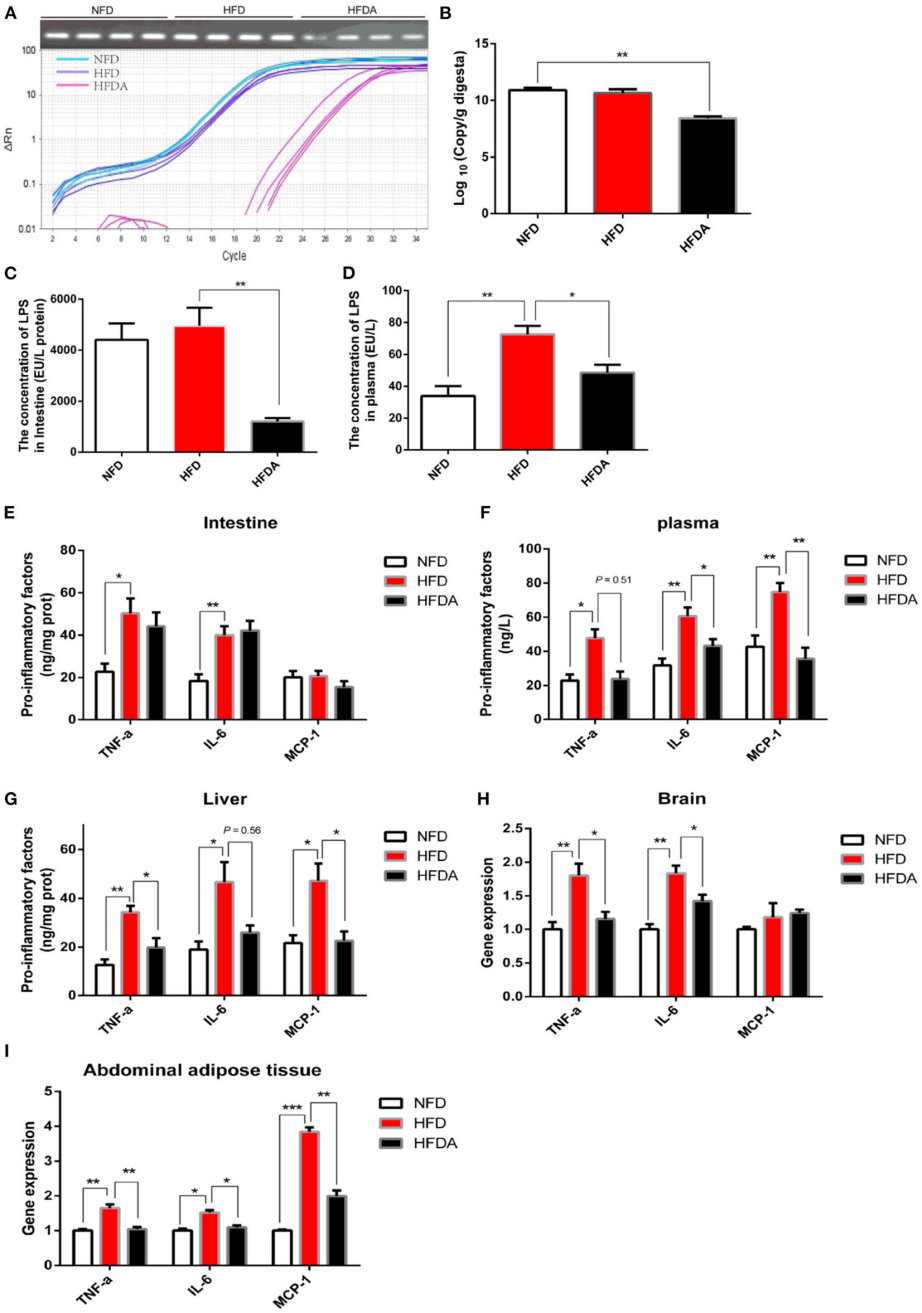
Antibiotic interference inhibits the penetration of LPS and then alleviates chronic inflammation induced by high fat. Fish were fed a normal-fat diet (5% fat, NFD), a high-fat diet (10% fat, HFD) or a high-fat diet supplemented with antibiotic (vancomycin 2 g/kg, erythromycin 4 g/kg, neomycin sulfate 4 g/kg and metronidazole 4 g/kg, HFDA) for 12 weeks. **(A)** Abundance analysis of total bacteria: agarose gel electrophoresis and PCR amplification curve. **(B)** Quantitative analysis of total bacteria. **(C,D)** The content of LPS in intestine **(C)** and plasma **(D)**. **(E–G)** The protein content of pro-inflammatory factors (TNF-α, IL-6 and MCP-1) in the intestine **(E)**, plasma **(F)** and liver **(G)**. **(H,I)** Gene expression of pro-inflammatory factors in brain **(H)** and abdominal fat tissue **(I)**. Data are shown as mean ± SEM. *N* = 4. Two-tailed unpaired Student's *t*-test was used. *P < 0.05, **P < 0.01, ***P < 0.001.

To explore whether the penetration of LPS was an initial inducement of low-grade chronic inflammation caused by high-fat diets, antibiotics were administrated to inhibit the production of LPS by suppressing the microbial diversity, especially for gram-negative bacteria. The number of total bacteria was significantly suppressed after antibiotic intervention (Figures 2A,B). In order to specifically decrease the production of LPS, we mixed vancomycin and erythromycin in the antibiotic interference to suppress the abundance of gram-negative bacteria. As expected, antibiotic intervention succeeded to reduce the production of LPS in the intestines (Figure 2C). Logically, we found that the leakage of LPS into the blood circulation due to excessive fat was reversed after antibiotics were treated (Figure 2D). Next, we verified the hypothesis that LPS was the initial cause of chronic inflammation induced by high fat when LPS production and its penetration were inhibited. Inconsistent with our expectations, the release of pro-inflammatory factors (TNF-α and IL-6) in the intestinal tissue failed to be alleviated after inhibiting the penetration of LPS (Figure 2E). With reference to previous studies, we realized that intestine damage and inflammation possibly was induced after the intestinal cavity was long-term exposure to antibiotics (28, 29). The content of intestinal inflammatory factors was comprehensively regulated by the level of dietary lipid and antibiotics in the HFD with antibiotics. For the intestinal organs, the alleviation of the inhibition of LPS on chronic inflammation might be interfered by antibiotic-induced intestinal injury. To continuously verify our hypothesis, inflammation was assessed in systemic circulation and the liver. Consistent with our hypothesis, consumption of HFD significantly increased the protein content of TNF-α and IL-6 in the liver and plasma, while this physiological phenomenon was reversed when the penetration of LPS was prevented (Figures 2F,G). Logically, inhibition of LPS penetration appeased inflammation-mediated immune response, which was evidenced by a significantly reduced protein content of MCP-1 (Figures 2F,G). To further verify this result, the gene expression of inflammatory factors in brain tissue and abdominal fat was tested. Consistent results were observed that the gene expression of pro-inflammatory factors was significantly reduced after LPS penetration was suppressed (Figures 2H,I). Together, these data demonstrated that obesity-related chronic inflammation induced by HFD was attributed to the penetration of LPS into the systemic circulation.

### High-Fat Diet Increases Intestinal Permeability and Then Weaken the Interception of LPS

**Figure 3:**
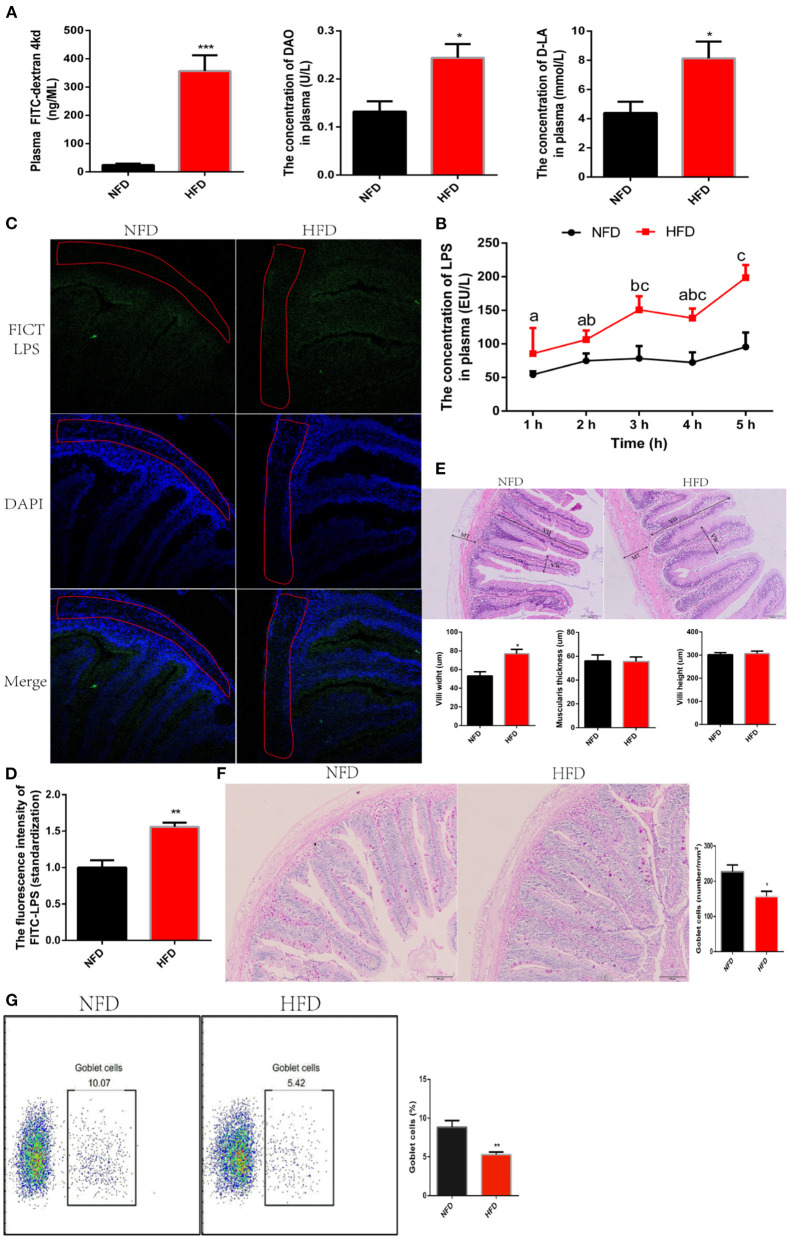
High-fat diets increase intestinal permeability and then weaken the interception of LPS. **(A)** Parameters of intestinal permeability: pasma FITC-d, D-LA and DAO. Plasma FITC-d was measured at 4 hours after oral administration of (900 mg/kg) FITC-d. **(B)** Assessment of LPS penetration into the circulation. Real-time fluctuation of plasma LPS concentration is monitored within 1–5 h after oral administration of (1 mg/kg) LPS. **(C)** Observation of LPS migration in the intestine using confocal laser at 5 hours after oral administration of (1 mg/kg) FITC-LPS. **(D)** Analysis of FITC-LPS concentration in plasma using fluorescence microplate reader for at 5 h after oral administration of (1 mg/kg) FITC-LPS. **(E)** Histology analysis of intestinal by hematoxylin-eosin (HE) staining. **(F)** Histology analysis of goblet cells by periodic acid-schiff (PAS) stain. **(G)** The number of goblet cells was counted with a flow cytometer. Data are shown as mean ± SEM. *N* = 4. Two-tailed unpaired Student's *t*-test was used. *P < 0.05, **P < 0.01, ***P < 0.001; One-way ANOVA was used. Diverse letters show significant differences (*P* < 0.05).

LPS are fenced in the intestinal cavity due to the defense of an integrated intestine barrier. LPS translocate from the gut into the systematic circulation and then induce inflammation when intestinal barrier is compromised (30). Now, we had confirmed interference with antibiotics to inhibit the production of LPS alleviated low-grade chronic inflammation. Unfortunately, antibiotics cannot be treated for a long time because of its resistance and irritation to the intestines Therefore, we further understood the mechanism of LPS crossing the intestine into the systemic circulation, which would provide a theoretical basis for formulating treatment strategies. To answer this question, we evaluated the effect of HFD on intestinal permeability. After feeding HFD for 12 weeks, fluorescein isothiocyanate dextran (FITC-d, Molecular weight = 4,000 kd) was orally administrated to evaluate intestinal permeability. We found that consumption of HFD significantly increased the concentration of FITC-d migrating to blood circulation, which showed that the intestinal permeability was increased (Figure 3A). Once the intestinal permeability increases, exogenous metabolites, such as diamine oxidase (DAO) and D-lactate (D-LA), migrate to the serosal layer of the intestine and subsequently enter systemic circulation (31). Consistent with increased intestinal permeability, the concentration of DAO and D-LA in plasma increased significantly after exposure to HFD for 12 weeks (Figure 3A). To explore whether the increase in intestinal permeability induced by HFD could facilitate LPS to escape the intestinal barrier and migrate into the systematic circulation, we detected the dynamic fluctuation of LPS in plasma after oral administration of 1 mg/kg LPS. Data showed that the intestinal barrier in the NFD group effectively blocked the penetration of LPS into the circulation. At the same time, the consumption of HFD caused the failure of the intestinal barrier to restrict LPS and significantly increased the penetration of LPS into the circulation (Figure 3B). We could not be sure that the increased LPS in plasma was sourced from oral administration of exogenous LPS because it possibly was caused by endogenous aggregation. To solve this problem, LPS was labeled with fluorescence using FITC and then was orally administrated. The distribution of LPS-FITC migrating to the intestine was observed with a laser confocal, and its content penetrating into the blood circulation was detected with a fluorescence microplate reader. We found that consumption of HFD increased the content of LPS-FITC migrating to muscular thickness (Figure 3C). The plasma fluorescence intensity value excited by LPS-FITC in the HFD group was significantly higher than that in the NFD group (Figure 3D). These results indicated that the increase in intestinal permeability induced by HFD could allow LPS to cross the intestinal barrier and enter into the systemic circulation. Next, we investigated predisposing factors underlying the increased intestinal permeability. An increased intestinal permeability was a trend to be accompanied by intestinal damage. We observed that in the organizational structure of intestine. No obvious structural damage was found, and while a significantly increase in villi width was found in the HFD group (Figure 3E). This may be an adaptive strategy to increase the intestinal area to digest and absorb excessive lipid. Goblet cells are specialized secretory cells and play an important role in maintaining the intestinal barrier by secreting a variety of factors, which contribute to the mucus layer protecting the mucosal epithelium. In this study, we found that the consumption of HFD significantly reduced the number of GCs via seriodic acid-schiff (PAS) stain (Figure 3F). To further verify this data, the number of GCs was measured by flow cytometry after it was labeled with MUC2 antibody. Consistent results showed that the number of GCs in the HFD group was significantly lower than that in the NFD group (Figure 3G). Together, the increased intestinal permeability induced by HFD allows LPS to penetrate the blood circulation.

### High-Fat Diet Weakens Mucosal Barrier via Disturbing the Secretion of MUC2 in Goblet Cell

**Figure 4:**
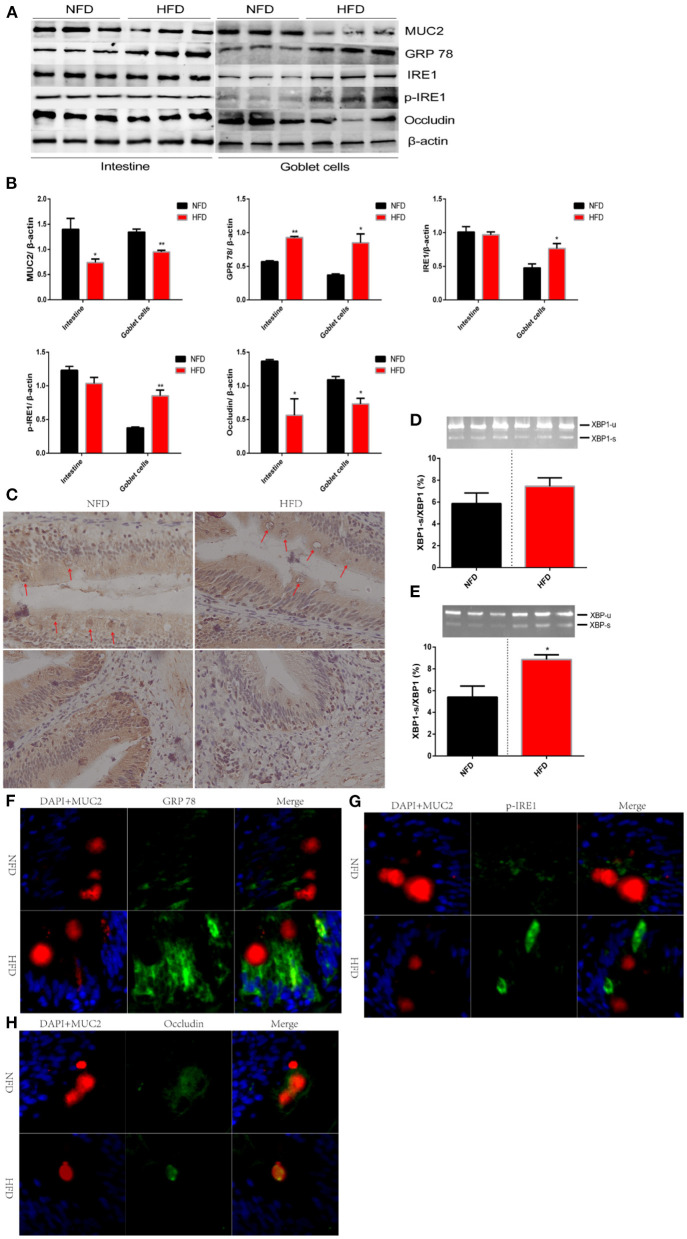
High-fat diets increase intestinal permeability via disturbing the secretion of MUC2 in goblet cell. Fish were fed a normal-fat diet (5% fat, NFD) or high-fat diet (10% fat, HFD) for 12 weeks. **(A)** polyacrylamide gel electrophoresis (SDS-PAGE) of MUC2, GPR 78, IRE1, p-IRE1 and occludin in intestinal tissue or isolated goblet cells. **(B)** Quantitative western blot analysis of MUC2, GPR 78, IRE1, p-IRE1 and occludin in intestinal tissue or isolated goblet cells. **(C)** Immunohistochemical analysis for the distribution of MUC2 in the intestinal tissue. Goblet cells are marked with red arrows. **(D,E)** The ratio of spliced to unspliced XBP1 mRNA in ntestinal tissue **(D)** or isolated goblet cells **(E)**. **(F–H)** Immunofluorescence analysis for the relocalization of GPR 78, p-IRE1 and occludin. The nucleus is stained by DAPA (blue), goblet cells is stained by MUC2 (red) and GPR 78 **(F)**, p-IRE1 **(G)** and occludin **(H)** is stained by corresponding antibody (green). Data are shown as mean ± SEM. *N* = 4. Two-tailed unpaired Student's *t*-test was used. *P < 0.05, **P < 0.01.

Intestinal mucosal barrier, an integral mucous layer, isolates intestinal epithelial cells from the harsh environment of the intestinal cavity. MUC2, an exocrine protein biosynthesized in GCs, locates at the mucosal layer to form a polymer skeleton (32). Intestinal homeostasis was disturbed and intestinal barrier was weakened when the MUC2 protein was knocked out or inhibited (33). The insufficient secretion of MUC2 has been correlated with numerous pathophysiological mechanisms, influencing several inflammatory diseases (34, 35). We had found a reduced number of GC (globlet cell) after exposure to HFD. Next, we explored whether the secretion of MUC2 protein was inhibited with the decreased number of GCs or was compensated through negative feedback. In this study, we found that consumption of HFD significantly inhibited the secretion of MUC2 protein compared with that in NFD (Figures 4A,B). The distribution of MUC2 protein in the intestinal tissue was observed via immunohistochemistry. Results showed that the abundance of MUC2 protein in the muscularis base was reduced after HFD was administered. In addition, prominent vacuoles were observed in GCs, which indicated an inhibited synthesis of MUC2 (Figure 4C). Exocrine protein was synthesized, maturated and secreted in the endoplasmic reticulum (ER) membrane (36). When the homeostasis of the internal environment is disturbed by the excessive lipid, proteins destined for secretion cannot be adequately assembled and then ER stress is induced (37). Glucose regulated protein 78 (GRP78) is a molecular chaperone protein residing in ER. GPR 78 is quickly activated as response strategies to eliminate misassembled proteins, and therefore is considered as a marker protein of ER stress (38). In this experiment, we found that the consumption of HFD significantly increased the protein abundance of GPR78 (Figures 4A,B). GPR78 initiated a series of response strategies to ER stress by activating the inositol-requiring enzyme 1 (IRE1) /X box-binding protein 1 (XBP1) signal pathway. GRP 78 dissociates from IRE1, the latter induces the unconventional splicing of XBP1, and finally spliced XBP1 enters the nucleus and inhibits the transcription of exocrine proteins to prevent more unfolded protein (39). IRE1-XBP1 pathway plays an important role in down-regulating the transcription of MUC2 to optimize mucin protein production (40). Unexpectedly, our results showed that the protein expression of IER1 showed no significant difference between the HFD group and NFD group (Figures 4A,B). Furthermore, we tested the protein level of p-IRE1 and the unconventional splicing of XBP1, both of which showed no significant difference between the two groups (Figures 4A,B,D). MUC2 is secreted by GCs. Secretory cells, such as islet cells, show poor resistance to high fat because multiple negative effects, including endoplasmic reticulum stress, have been reported in high-fat models (41, 42). We speculated that the IRE1/XPB-1 pathway might be activated explicitly in GCs. We used a flow cytometry to sort out GCs after it was marked by the MUC2 antibody. Consistent results were observed in GCs that the protein expression of MUC2 was significantly suppressed and while the protein expression of GPR 78 was significantly enhanced after HFD was administered for 12 weeks (Figures 4A,B). As our hypothesis, IRE1/XPB-1 pathway was activated, which was evidenced by an increase in protein expression of IRE1 and p-IRE1 as well as mRNA abundance of the unconventional splicing of XBP1 (Figures 4A,B,E). To further verify this conclusion, we used immunofluorescence to observe the distribution of GPR and p-IRE1 after GCs were marked by MUC2 (Figures 4F,G). Consistent results were shown that consumption of HFD increased the distribution of GPR78 and pIRE1 proteins in GCs. Occludin is mainly located in the cell membrane and protects secretory cells from ER stress (43). In this experiment, we found that the protein expression of occludin in the intestine and isolated GCs was significantly reduced due to excessive dietary lipid (Figures 4A,B). Immunofluorescence results showed that occludin in NFD was mainly distributed in the cell membrane and while that in HFD migrated to the cytoplasm (Figure 4H).

### Excessive Lipid Facilitates the Transfer of LPS Across Intestinal Cell Barrier

**Figure 5:**
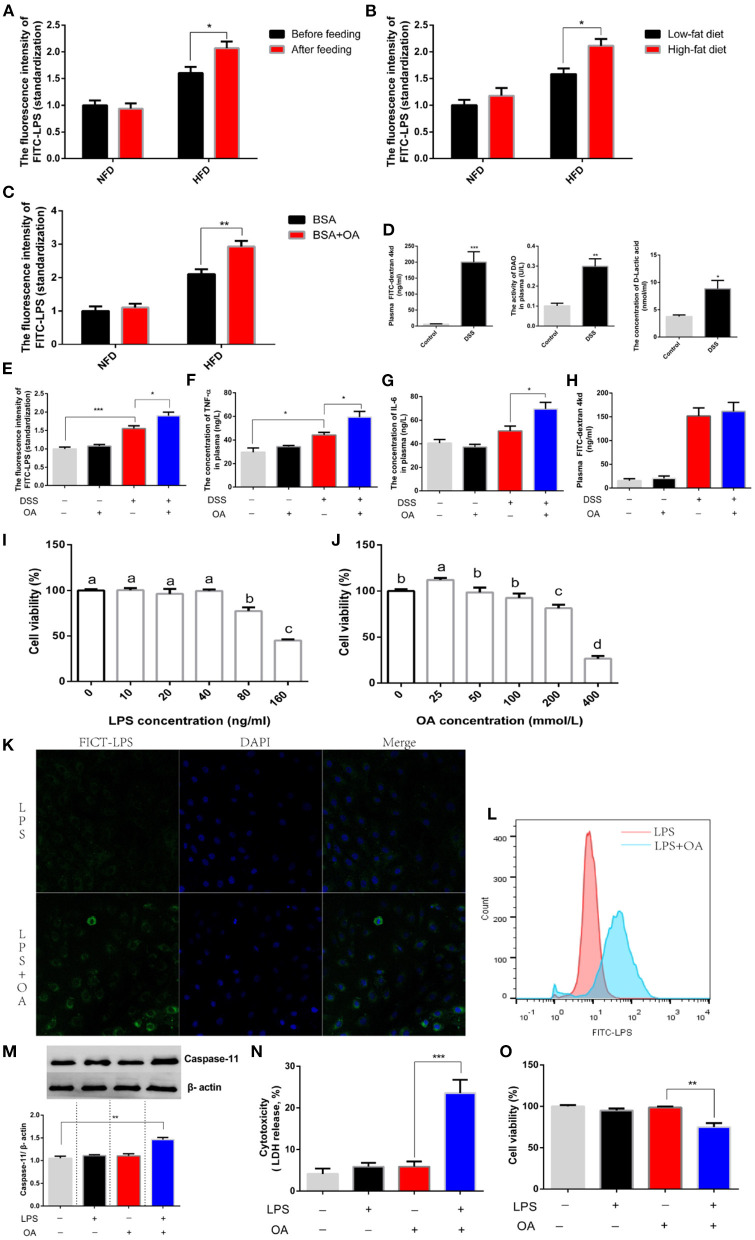
Fatty acids assist the transfer of LPS. **(A–C)** The concentration of plasma FITC-LPS in fish were fed an NFD or HFD. **(A)** The analysis was performed in two groups (NFD and HFD) before or after feeding. **(B)** The analysis was performed in two groups (NFD and HFD) after feeding Low-fat diet (5% fat) or high-fat diet (10% fat). **(C)** The analysis was performed in two groups (NFD and HFD) was simultaneously gavage BSA or BSA mixed with OA. **(D)** Evaluation of intestinal permeability after DSS was fed. **(E-H)** Fish fed with diets (free or supplement with DSS) was orally administered with FITC-LPS in the presence or absence of OA. **(E)** The concentration of plasma FITC-LPS. **(F)** The concentration of plasma TNF-α. **(G)** The concentration of plasma IL-6. **(H)** The concentration of plasma FITC-d. **(I,J)** The analysis of cell viability after IEC-6 is incubated with different levels of LPS LPS or OA. **(K)** Observation of the intracellular migration of FICT-LPS in the presence or absence of OA using laser confocal. **(L)** Analysis of intracellular FICT-LPS using flow cytometer. (M-O) IEC-6 was incubated with LPS, OA or LPS mixed OA for 24h. **(M)** Western blot analysis of caspase 11. **(N)** Cytotoxicity. **(O)** cell viability. Data are shown as mean ± SEM. *N* = 4. Two-tailed unpaired Student's *t*-test was used. *P < 0.05, **P < 0.01, ***P < 0.001; One-way ANOVA was used. Diverse letters show significant differences (*P* < 0.05).

Next, we explored the mechanism by which LPS escaped the epithelial cell barrier. Interestingly, we discovered that feeding the corresponding diets before oral administration of LPS significantly increased LPS penetration (Figure 5A). This result hinted an existence of nutrients in the diet promoted the migration of LPS. Logically, we guessed that dietary lipid levels might contribute to the increased penetration of LPS. To further explore this issue, diets supplemented with different grades lipid (5 and 10% lipid) was fed before oral administration of LPS. We found that high-grade dietary lipid significantly increased the penetration of LPS (Figure 5B). Further, we compared the penetration of LPS after oral administration of LPS in the presence or absence of oleic acid (OA), a monounsaturated fatty acid. A significant increase in LPS penetration was observed when LPS was treated with OA (Figure 5C). However, feeding status, dietary fat and fatty acids showed no effect on LPS penetration in experimental animals provided NFD. Given the above results, we believed that lipid possibly assisted LPS in escaping the intestinal barrier when intestinal permeability was increased. To verify this hypothesis, an intestinal-injury model was established where intestinal permeability was increased by feeding a diet supplemented with dextran sulfate sodium (DSS). After DSS was treated for 15 days, the intestinal permeability was tested by oral administration of FICT-d. A significant increase in plasma FICT-d was detected due to DSS administration, which demonstrated an increased intestinal permeability (Figure 5D). Consistently, a significant increase in the concentration of plasma DAO and LA was shown in animal fed with diets supplemented DSS (Figure 5D). Based on this model, we explored whether lipid was capable of assisting the migration of LPS. We found that the concentration of LPS penetration in the presence of OA was significantly higher than that in the absence of OA (Figure 5E). Consistently, the inflammatory response was exacerbated after oral administration of LPS in the presence of OA, which was supported by the significantly increased release of TNF-α and IL-6 in plasma (Figures 5F,G). The same conclusion was verified that lipid increased the transfer of LPS, while this phenomenon was not observed in the control group when the intestinal barrier was integrated. We believed that short-term (4h) oral administration of lipid did not increase intestinal permeability. In fact, we found that the intestinal permeability was not increased after OA was administered, which was supported by no significant difference in plasma FICT-d (Figure 5H). A non-canonical inflammasome was found after LPS was recognized by intracellular signaling molecules, which showed that LPS could be transferred into the cytoplasm. Therefore, we speculated that lipid possibly facilitated the transfer of LPS into the cytoplasm and then escaped the intestinal cell barrier. Intestinal epithelial cell line (IEC-6) was used as a tool to explore this issue. The concentration of LPS lower than 40 ng/ml and OA lower than 100 mmol/L was regarded as a non-toxic dose according to those effects on cell viability (Figures 5I,J). We observed the migrating of LPS into the cytoplasm using a flow cytometer and found the number of cells infected with FICT-LPS increased significantly in the presence of OA (Figure 5K). The same result also was observed using a laser scanning confocal microscope that OA improved the internalization of LPS into the intracellular environment (Figure 5L). The LPS migrating into the cytosol would be sensed by caspase-11 and activated intracellular signaling pathways to induce injury. We found that the protein abundance of caspase-11 significantly increased after intracellular LPS was increased by OA (Figure 5M). Consistent with the activation of LPS-mediated intracellular signaling pathways, cytotoxicity was significantly increased (Figure 5N) and cell viability was significantly inhibited (Figure 5O). In short, lipid facilitated LPS penetration by increasing their cellular internalization.

### Excessive Lipid Facilitates the Transfer of LPS Depending on CD36

**Figure 6:**
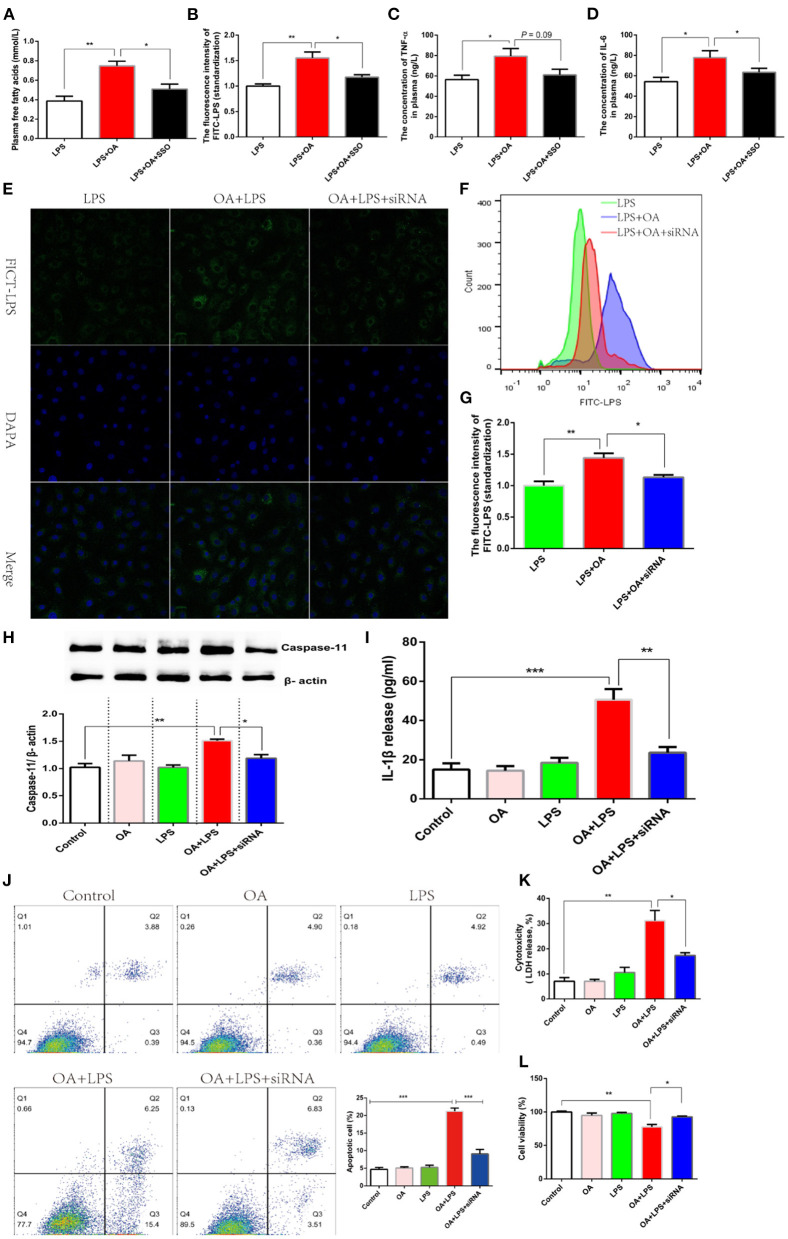
Fatty acids facilitate the transfer of LPS through CD36. **(A–D)** Fish was orally administered with FITC-LPS, FITC-LPS mixed with OA, or FITC-LPS mixed with OA and SSO (CD36 inhibitor) after a diet supplemented with DSS is treated for 15 days. **(A)** The concentration of plasma FITC-LPS. **(B)** The concentration of plasma FITC-d. **(C)** The concentration of plasma TNF-α. **(D)** The concentration of plasma IL-6. **(E–G)** IEC-6 was incubated with FITC-LPS, FITC-LPSmixed with OA, or FITC-LPS mixed with OA and siRNA CD36. **(E)** Observation of the intracellular migration of FICT-LPS using laser confocal. **(F)** Analysis ofintracellular FICT-LPS using flow cytometer. **(G)** Qalitative analysis of intracellular FICT-LPS using fluorescence microplate reader. **(H-L)** IEC-6 was incubated withFITC-LPS, OA, FITC-LPS mixed with OA, or FITC-LPS mixed with OA and siRNA CD36. **(H)** Western blot analysis of caspase 11. **(I)** Release of IL-1β. **(J)** Flowcytometry for analysis ofapoptosis. **(K)** Cytotoxicity. **(L)** Cell viability. Data are shown as mean ± SEM. *N* = 4. Two-tailed unpaired Student's *t*-test was used. ^*^P < 0.05, ^**^P < 0.01, ^***^P < 0.001.

Deacylation of lipid A, a similar structure with long-chain fatty acids, blocks the intracellular signaling pathway mediated by LPS (12). Fatty acid translocase (FAT, CD36), a transmembrane protein, is responsible for the extradition of fatty acids across the cell membrane (44). Another independent experiment showed an increased CD36 expression after mammary gland epithelial cells were incubated with LPS (45). Therefore, we hypothesized that lipid assisted LPS to transfer into an intracellular environment depending on the activating of CD36. Firstly, Sulfo-N-succinimidyl Oleate (SSO) was introduced as an inhibitor of CD36 to verify this hypothesis in the model where intestinal permeability was increased by DSS. 50 mg/kg SSO inhibited CD36 via binding its lysine 164, which was tested by the transfer efficiency of free fatty acids (46). We found that oral gavage of 50 mg/kg SSO significantly inhibited plasma free fatty acids, which supported a suppressed CD36 (Figure 6A). OA significantly increased the quantity of LPS penetrating into plasma, and while this effect was reversed after CD36 inhibitor was treated (Figure 6B). Consistently, the higher-grade release of pro-inflammatory factors caused by LPS penetration were also significantly inhibited after CD36 inhibitor was treated (Figures 6C,D). These data suggested that OA promoted the penetration of LPS depending on the activation of CD36. Considering the the non-specific inhibition of chemical inhibitors, we verified this conclusion in IEC-6 cell lines using siRNA CD36. LPS migrating into the cytoplasm promoted by OA was inhibited after siRNA CD36 was incubated (Figure 6E). The results of flow cytometry analysis also showed that OA increased the number of cells with high fluorescence excitated by FICT-LPS, and this biological effect was eliminated when CD36 was inhibited (Figure 6F). Further, the cytoplasmic content of LPS was detected by a fluorescent microplate reader, and the same result was observed that siRNA CD36 suppressed the migration of LPS to the cytoplasm (Figure 6G). Since CD36 hindered the migration of LPS to the intracellular environment, it was logical that the intracellular signaling pathway mediated by LPS was also blocked. Intracellular LPS activated non-canonical inflammasome dependent of caspase-11 and then promoted the release of IL-1β. As expected, the inhibit of CD36 blocked the intracellular pathway of LPS enhanced by OA, which was evidenced by a significant reduction in the protein abundance of caspase-11 and the release of IL-1β (Figures 6H,I). After cytoplasmic LPS was recognized by caspase-11, a form of programmed cell death was enabled to defend against invading. In this study, we found that LPS-induced apoptosis in the presence of OA was significantly inhibited after siRNA CD36 was treated (Figure 6J). Consistent with apoptosis, LPS in presence of OA significantly suppressed cell viability and enhanced cytotoxicity, which is reversed after CD36 was inhibited (Figures 6K,L).

## Discussion

With the popularity of the modern western diet composed of foods rich in fat, obesity has achieved the shape of a pandemic worldwide and poses a threat to the health of 650 million people (1). Obesity increases the risk of diverse pathologies, such as non-alcoholic fatty liver disease, certain types of cancers, type 2 diabetes, and microvascular diseases. Obesity is characterized by persistent and low-grade inflammation in multiple organizations, such as the hypothalamus (47), adipose tissue (48), liver (49) and intestine (50). We all know that inflammation is an important chain transmission of initiating an immune response. However, the long-term release of inflammatory factors will over-activate the immune defense and eventually cause autoimmune damage. Our previous research found that high-fat-induced inflammation plays an irreplaceable role in the development of apoptosis, DNA damage, immune cell infiltration, liver fibrosis (20). Relieving high-fat-induced inflammation may be a target site for preventing obesity-related diverse pathologies. So far, the molecular origin of low-grade inflammation is controversial. In this experiment, we found that the leakage of LPS infiltrating into the systematic circulation was a potential inducement for chronic inflammation. In addition, we found for the first time that the leakage of LPS was caused by the disturbance of the intestinal mucosal barrier due to insufficient secretion of MUC2. Further, lipid assisted the transfer of LPS into cytosol depending on activating CD36, which might be the mechanism by which LPS escapes the intestinal epithelial barrier.

In this study, we established a high-fat model using *M. amblycephala* where similar obesity-related pathologies reported in mammals were observed, such as unwanted depositions of lipid in the tissues, hyperglycemia, hyperlipidemia and increased abdominal fat. This high-fat model presented typical obesity-symptoms and therefore provided a suitable whole-body system for investigating obesity-related mechanisms. As observed in other high-fat models, obesity is accompanied by low-grade chronic inflammation, which is evidenced by significantly increased release of pro-inflammatory factors. Interestingly, we found that the LPS content in the blood increased significantly after exposure to high-fat. LPS is the main component of the outer membrane in gram-negative bacteria and is characterized as a typical pro-inflammatory biologically active substance. We hypothesized that the penetration of LPS was the molecular origin of chronic inflammation based on the following basis: (1) LPS penetrating into the circulation induced inflammation at very low concentrations. (2) A continuous subcutaneous low-rate infusion of LPS induced most, if not all, of the features of metabolic diseases. (3) Diverse nutritional interventions alleviated chronic inflammation by changing the composition of gut microbes, especially gram-negative bacteria (7–9). To verify this hypothesis, we used antibiotic intervention to suppress the total amount of intestinal microbes and then reduced the production of LPS. As per our expectations, consumption of HFD significantly increased the release of TNF-α, IL-6 and MCP-1, while these pro-inflammatory factors in multiple organizations were suppressed after the penetration of LPS was reduced by antibiotic intervention. Antibiotics cannot be administered for a long time because of drug resistance. Therefore, it is necessary to understand the mechanism of LPS penetration to provide a basis for practical treatment options. LPS is mainly derived from gram-negative bacteria and is trapped in the intestinal cavity by the defense of intestinal barrier. LPS translocates to the lymphatic system when intestinal permeability is increased due to disruption of the intestinal barrier. Intestinal permeability was significantly increased after high-fat diets were consumed, which had also been reported in mammals (10, 27). Here, we further investigated the mechanism of increased intestinal permeability. We observed the histological structure of the intestine and found no obvious damage. The mucosal layer protects the intestinal environment by separating colonized intestinal bacteria and therefore plays an important role in intercepting the migration of LPS. MUC2, the main component of mucus, is mainly secreted by goblet cells. Here, we found that consumption of HFD significantly reduced the number of goblet cells. Next, the distribution of MUC2 was observed with immunohistochemistry. The results showed that the distribution of MUC2 protein at the base of the intestine was reduced in experimental subjects eating HFD. In addition, a clear vacuole in the goblet cells was observed, indicating that the synthesis and secretion of MUC2 protein were blocked. Our previous research found that consumption of HFD blocked the maturation and transportation of the protein fated for secretion depending on ER stress mediated by IRE1/XBP1 signal pathway (37). This pathway also showed a predominant regulation of the secretion of MUC2 in goblet cells (40). Inconsistent with our expectations, we found that high fat increased the abundance of GPR78, a marker protein of ER stress, while the IRE1/XBP1 signal pathway was not activated. Previous research confirmed that secretory cells, typically such as pancreatic islet cells, were more susceptible to stress from high fat (41, 51). We suspected that the IRE1/XBP1 signaling pathway possibly was activated in goblet cells instead of the entire intestine. To verify this conjecture, we sorted out goblet cells with a flow cytometer and the IRE1/XBP1 signaling pathway was detected. As our supposed, an activated IRE1/XBP1 signaling pathway was observed in goblet cells after the consumption of HFD. Interestingly, we found that the protein abundance of occludin was suppressed in the intestine and goblet cells. Immunofluorescence analysis showed that occludin was mainly distributed in the cell membrane in the control group, while it in the high-fat diet group migrated to the cytoplasm. Occludin, a cell membrane protein, protects secreted cells against disturbances in the internal environment. Once occludin was knocked out, secretory cells were more prone to suffer from ER stress (43). Here we believed that the high-fat diet-induced ER stress might be due to the loss of the protective function of occludin. Unfortunately, the specific mechanism cannot be further explored because goblet cells cannot be cultured *in vitro* and occludin cannot be specifically knocked out in goblet cells in an animal model. In summary, the transfer of LPS into the blood circulation is the molecular origin of low-grade chronic inflammation. Mechanically, consumption of HFD weaken the intestinal mucus barrier by inhibiting the secretion of MUC2.

Intestinal epithelial cells (IECs) are bound close together by a series of tight junction protein to form a defensive barrier. It is generally accepted that exogenous toxic macromolecules, including LPS, dodge the IECs barrier via paracellular interspace when tight junction protein is disturbed. Here, we found that consumption of HFD increased LPS penetration via the transcellular pathway. First, we found that feeding corresponding diets before oral administration of LPS increased migration of LPS to the circulation in the HFD group, which implied the existence of nutrients promoting the migration of LPS. Finally, we determined that lipid assisted in the penetration of LPS. However, we did not observe this phenomenon in the control group. We speculated that an increased intestinal permeability was a prerequisite for lipid to promote the transfer of LPS. To further verify this, we induced intestinal damage by using DSS. Consistent results were observed that lipid promoted the efficiency of LPS migration into the blood circulation after the intestine was damaged, which was also supported by the significantly increased pro-inflammatory factors. Confusingly, we did not observe an increase in intestinal permeability after fatty acid was administered orally. FITC-d was introduced to assess intestinal permeability in this experiment. However, FITC-d was used limitedly as an indicator for assessment of intestinal paracellular permeability [52]. Therefore, we speculated that lipid possibly promoted the metastasis of LPS via the transcellular pathway. This conjecture was also supported by a fact that non-canonical inflammatory caspases were activated by cytosolic LPS, which showed that LPS could pass through the cell membrane into the cytoplasm and then transmitted between cells (17). The intestinal cell line was used as a tool to explore whether lipid promoted the cellular internalization of LPS. In this experiment, we found that the migration of LPS into the cytoplasm was increased in the presence of OA. Consistently, OA also activated the signaling pathway of intracellular LPS and therefore increased its cytotoxicity. Since the intracellular non-canonical pathway of LPS is found, most research has focused on key regulators involved in intracellular pathways. However, there is no report concerning how LPS crosses the cell membrane and enters the cytoplasm. Next, we explored the mechanism by which lipid promoted the cellular internalization of LPS. LPS are constituted by lipid A, core sugars, and the O-antigen. Lipid A is the most conserved molecule, and is mainly responsible for LPS bioactivity. Lipid A shares a similar structure with long-chain fatty acids. Fatty acids are absorbed into the cytoplasm by activating fatty acid transporters, which possibly facilitate the internalization of LPS. CD36, a cell membrane protein, is responsible for the extradition of extracellular fatty acids to the cytoplasm. More importantly, an independent research reported that the transcription level of CD36 was increased after LPS was treated (45). Therefore, we assumed that lipid activated CD36, which also assisted the penetration of LPS. SSO was introduced as a CD36 inhibitor. LPS migrating into the blood circulation is significantly inhibited after the CD36 inhibitor was treated. Consistently, the release of pro-inflammatory factors was also significantly suppressed. Furthermore, this mechanism was verified *in vitro*. We found that *in vitro* siRNA CD36 blocked the penetration of LPS into the cytoplasm and inhibited the intracellular pathway mediated by LPS. In summary, lipid increased the cellular internalization of LPS and its cytotoxicity depending on the activation of CD36. Protein carrier is necessary for long-chain fatty acids to transfer across the cell membrane. In order to transport fatty acids, CD36 is strategically increased, which facilitates the migration of LPS through lipid A. Non-canonical inflammasome mediated by the intracellular pathway of LPS has been reported, however the mechanism by which LPS crosses the cell membrane into the cytoplasm is not yet understood. Here, we found that lipid assisted the transmembrane of LPS dependence on CD36, providing a new target for defending against bacterial pathogens that invade the cytosol.

## Conclusion

**Figure 7:**
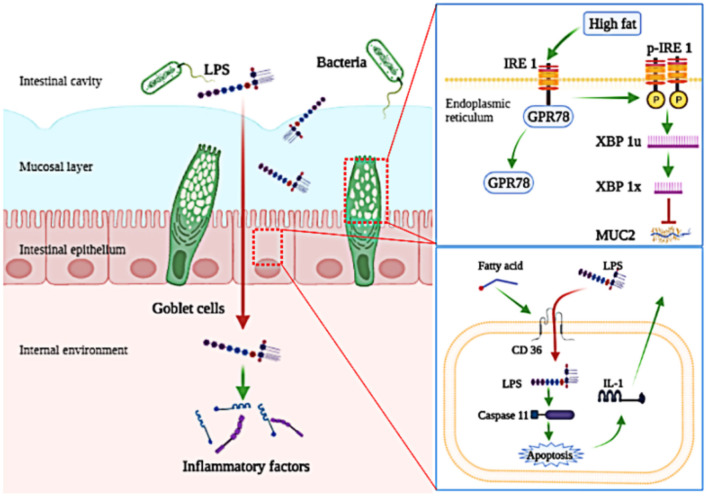
A schematic diagram shows key molecular pathways involved in the penetration of LPS induced by high-fat diet. Consumption of high-fat diets increases the penetration of LPS, which induces low-grade chronic inflammation; Consumption of high-fat diets inhibit the secretion of MUC2 by activating the IRE1/XPB-1 signaling pathway in goblet cells; Fatty acids assist in the cellular internalization of LPS and then activate its intracellular signaling pathway depending on CD36 in intestinal epithelial cells.

In conclusion, obesity-related inflammation is induced in *Megalobrama amblycephala* fed a HFD. Further, we find that the penetration of LPS into the blood circulation is the initial inducement of low-grade chronic inflammation induced by HFD. For precise mechanism, LPS escapes the mucosal barrier because consumption of HFD disturbed the secretion of MUC2; LPS escapes the intestinal epithelial cells barrier via a transcellular pathway with the assistance of CD36 (Figure 7).

## Data Availability Statement

The original contributions presented in the study are included in the article/**Supplementary Material**, further inquiries can be directed to the corresponding author.

## Ethics Statement

The animal study was reviewed and approved by Animal Care and Use Committee of Nanjing Agricultural University (Nanjing, China) (Permit Number: SYXK (Su) 2011-0036).

## Author Contributions

Y-JD designed and performed the experiments, analyzed the data, and wrote the manuscript. KX and XW performed the experiments and analyzed the data. W-BL and KA evaluated the data and participated in preparing the manuscript. D-DZ and X-FL provided suggestions for experiments and analyzed data. G-ZJ designed the experiments, provided suggestions for experiments, analyzed data, and wrote the manuscript. All authors contributed to the article and approved the submitted version.

## Funding

This research work was funded by the Shanghai Agriculture Applied Technology Development Program of China (G20190301), Jiangsu Natural Science Foundation for Basic Research (BK20201325), and National Technology System for Conventional Freshwater Fish Industries of China (CARS-45-14).

## Conflict of Interest

The authors declare that the research was conducted in the absence of any commercial or financial relationships that could be construed as a potential conflict of interest.

## Publisher's Note

All claims expressed in this article are solely those of the authors and do not necessarily represent those of their affiliated organizations, or those of the publisher, the editors and the reviewers. Any product that may be evaluated in this article, or claim that may be made by its manufacturer, is not guaranteed or endorsed by the publisher.
