# Supplementary figures and images for: The Mechanism of Lipopolysaccharide Escaping the Intestinal Barrier in Megalobrama amblycephala Fed a High-Fat Diet
Source: Front Nutr. 2022 Apr 7;9:853409. doi: 10.3389/fnut.2022.853409 (PMC9023073; doi:10.3389/fnut.2022.853409)

NFD


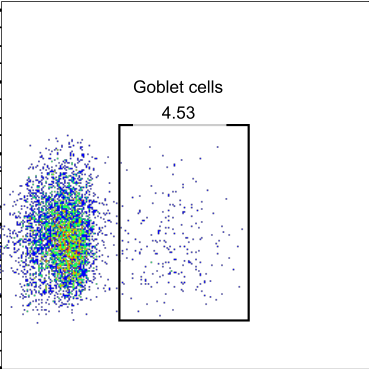

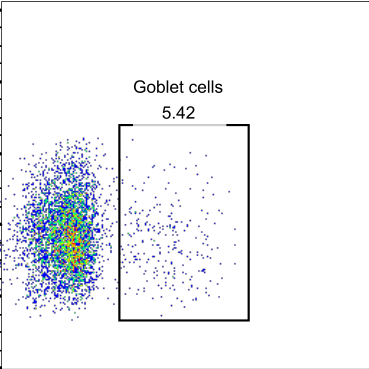

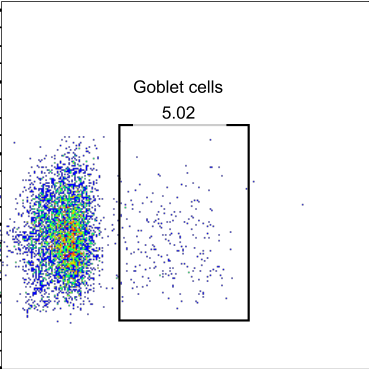

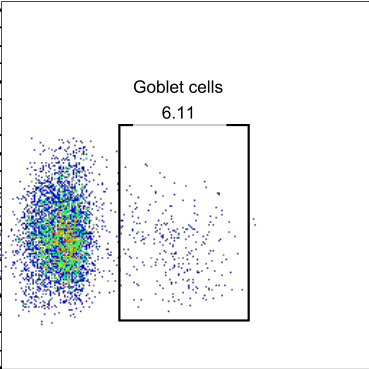


HFD


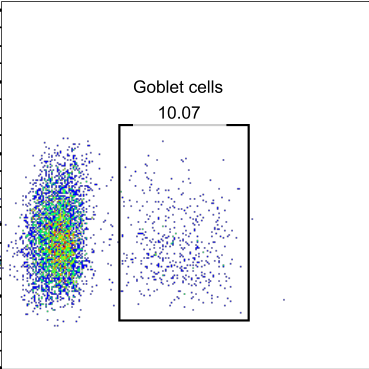

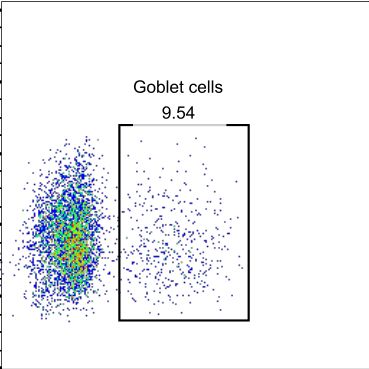

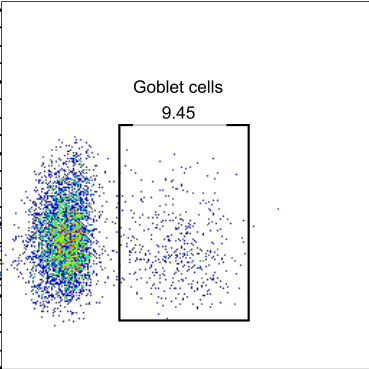

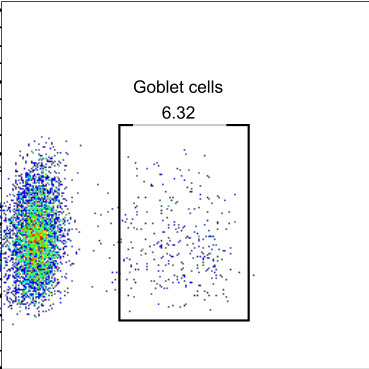

Supplement: Supplementary file 1 [file Data_Sheet_1.ZIP › raw data -/Fig. 3G .docx]

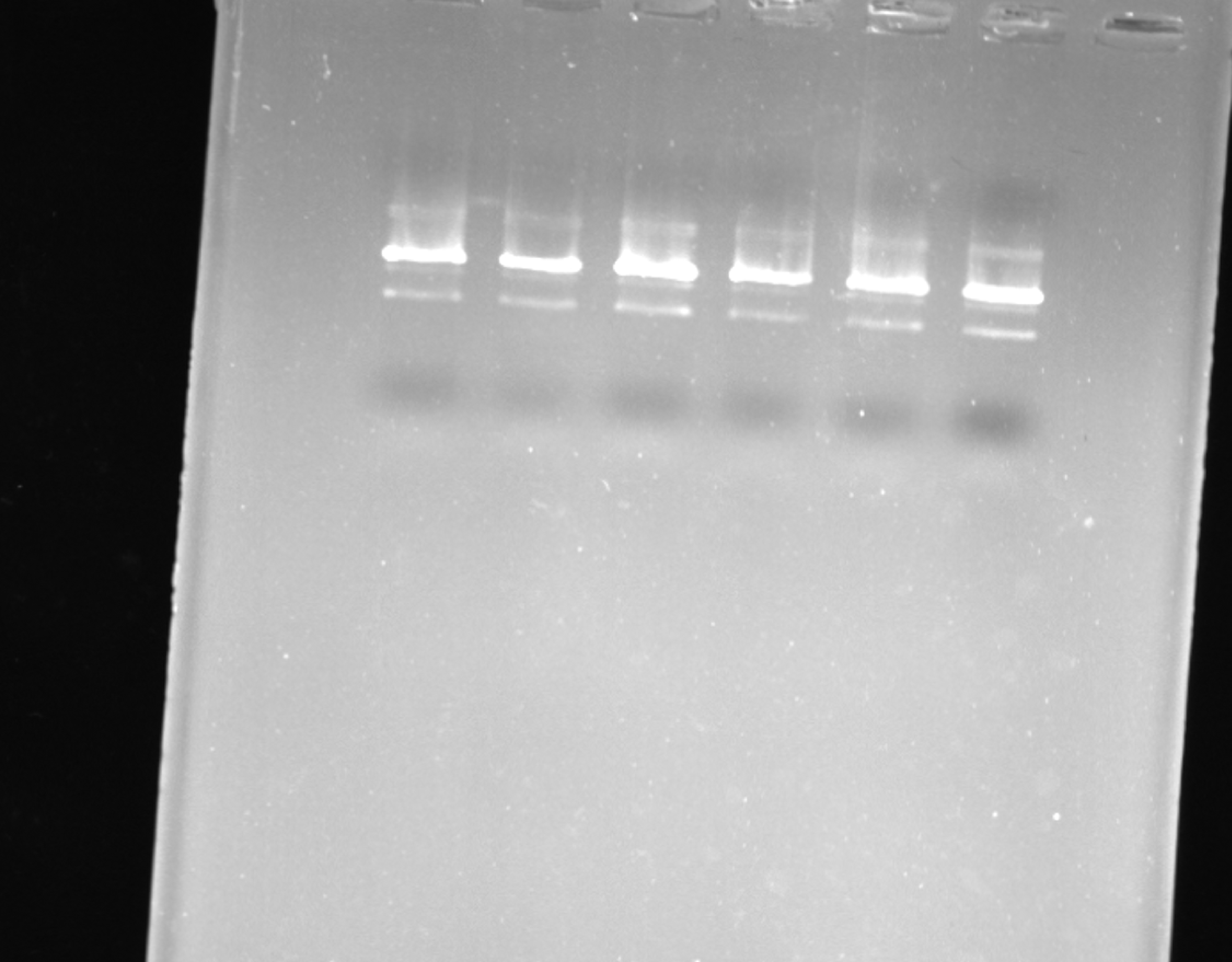

Supplement: Supplementary file 1 [file Data_Sheet_1.ZIP › raw data -/Fig. 4D.jpg]

C


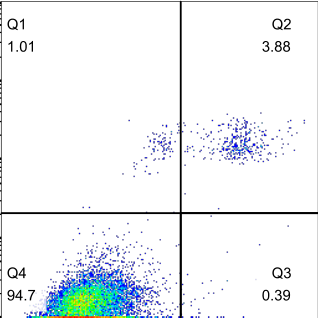

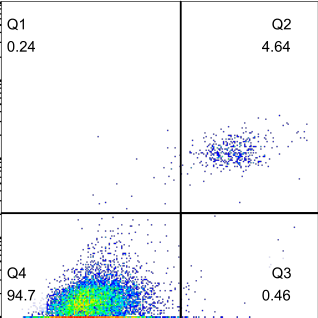


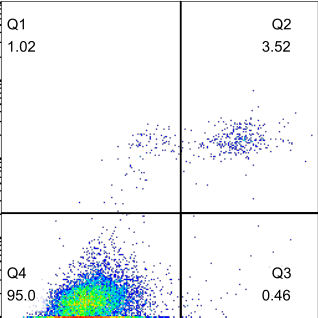

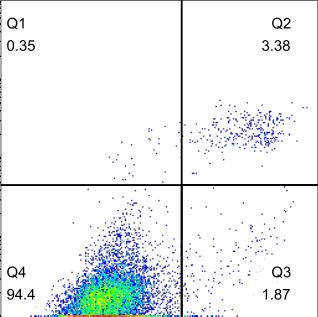


OA


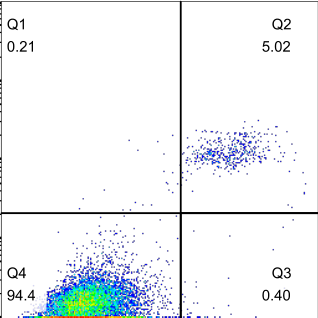

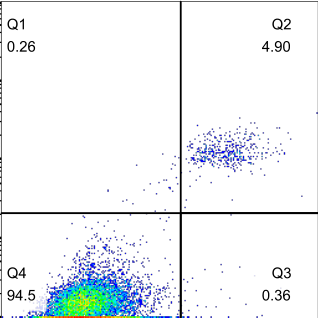


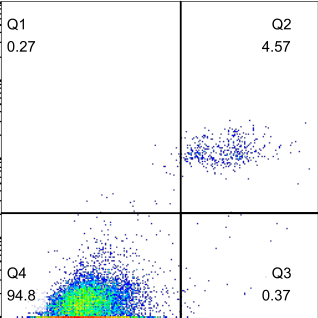

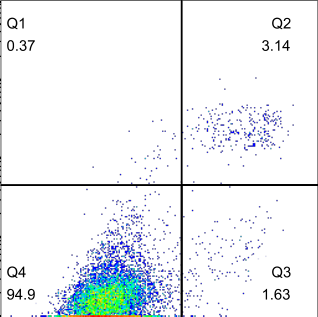


LPS


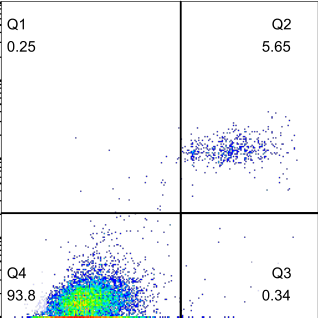

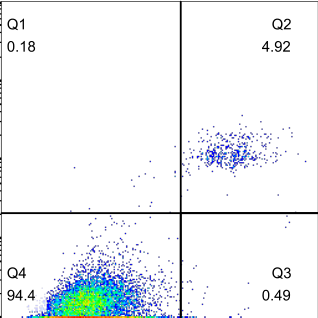


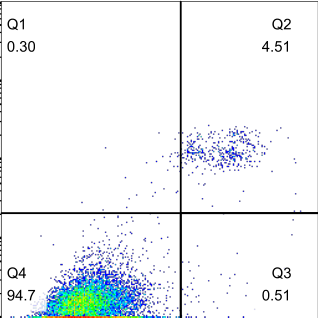

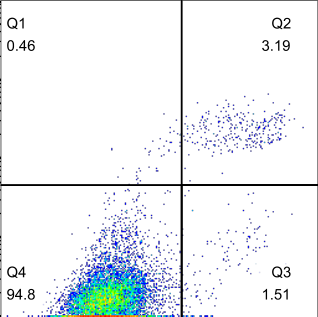


LPS+OA


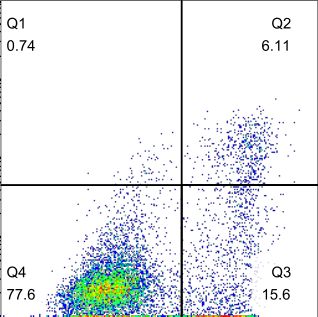

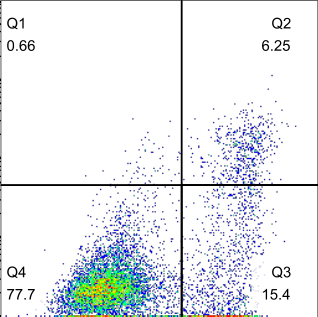


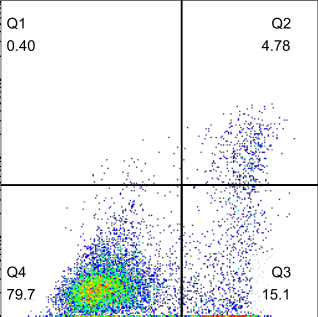

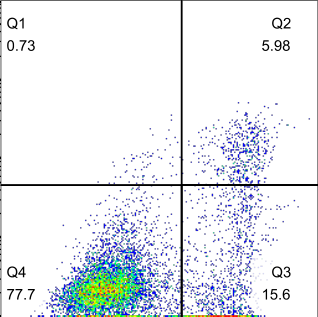


LPS+OA+siRNA


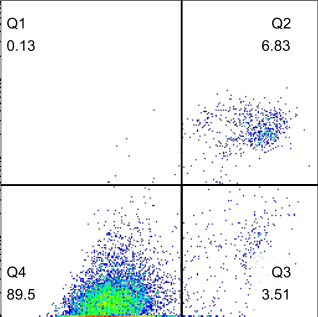

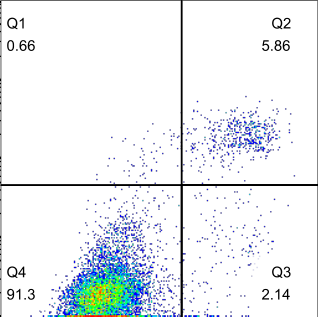


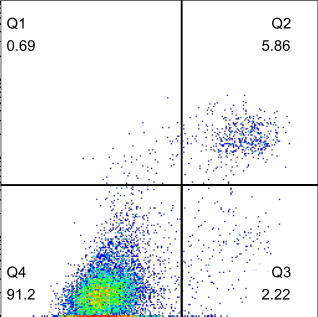

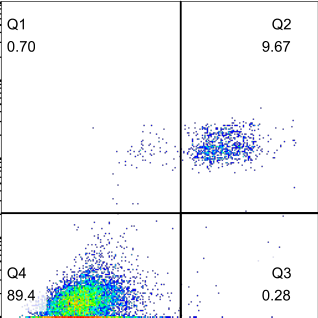

Supplement: Supplementary file 1 [file Data_Sheet_1.ZIP › raw data -/Fig. 6I.docx]

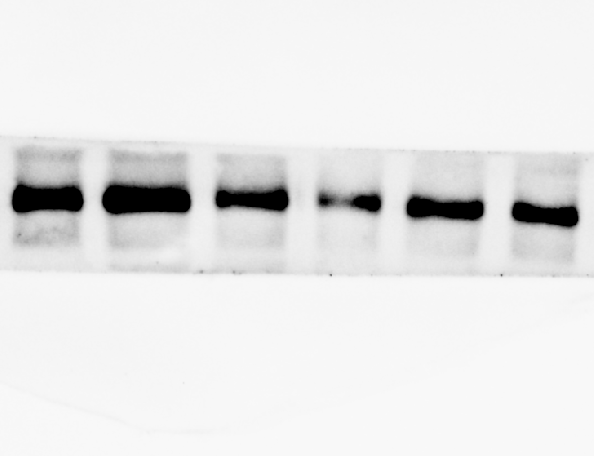

Supplement: Supplementary file 1 [file Data_Sheet_1.ZIP › raw data -/WB data/Fig. 2 MUC2-I.tif]

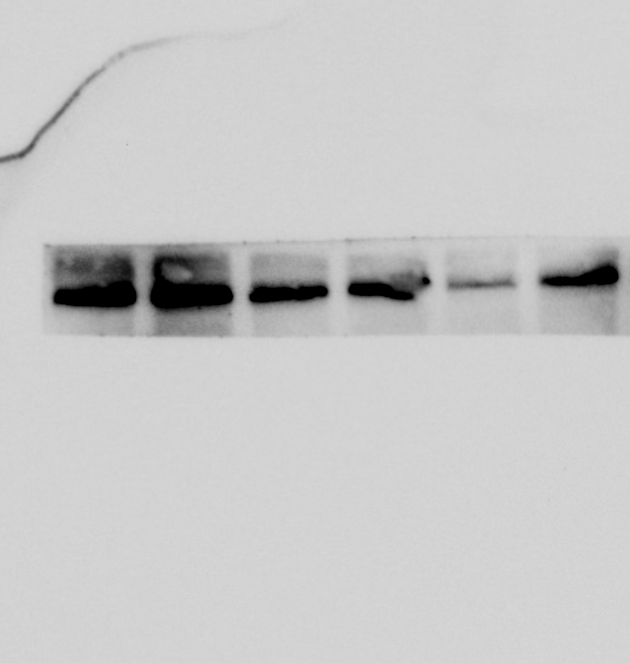

Supplement: Supplementary file 1 [file Data_Sheet_1.ZIP › raw data -/WB data/Fig. 2 occludin-GC.tif]

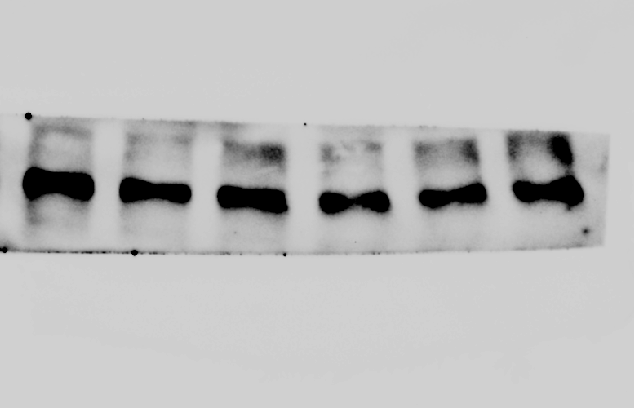

Supplement: Supplementary file 1 [file Data_Sheet_1.ZIP › raw data -/WB data/Fig. 2 occludin-I.tif]

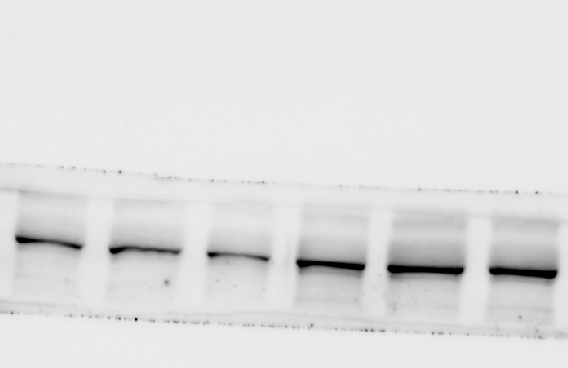

Supplement: Supplementary file 1 [file Data_Sheet_1.ZIP › raw data -/WB data/Fig. 2 pIRE1-GC.tif]

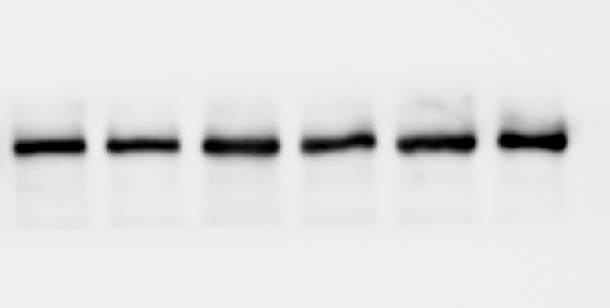

Supplement: Supplementary file 1 [file Data_Sheet_1.ZIP › raw data -/WB data/Fig. 2-action-I.tif]

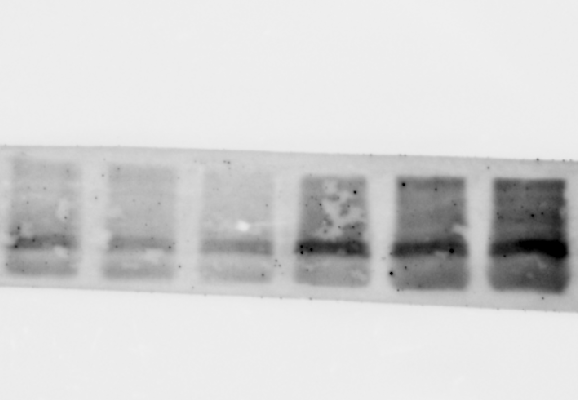

Supplement: Supplementary file 1 [file Data_Sheet_1.ZIP › raw data -/WB data/Fig. 2-p-IRE1-GC.tif]

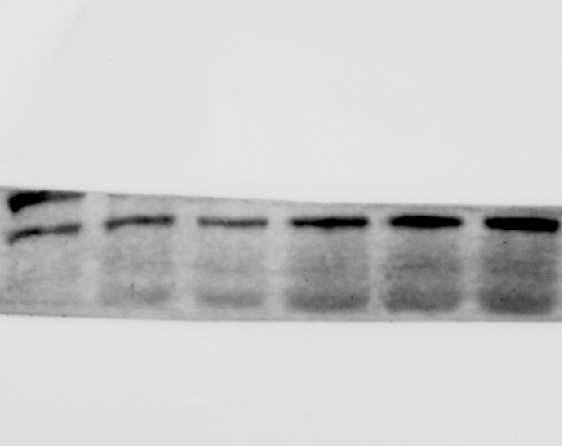

Supplement: Supplementary file 1 [file Data_Sheet_1.ZIP › raw data -/WB data/Fig. 2GPR-78-GC .tif]

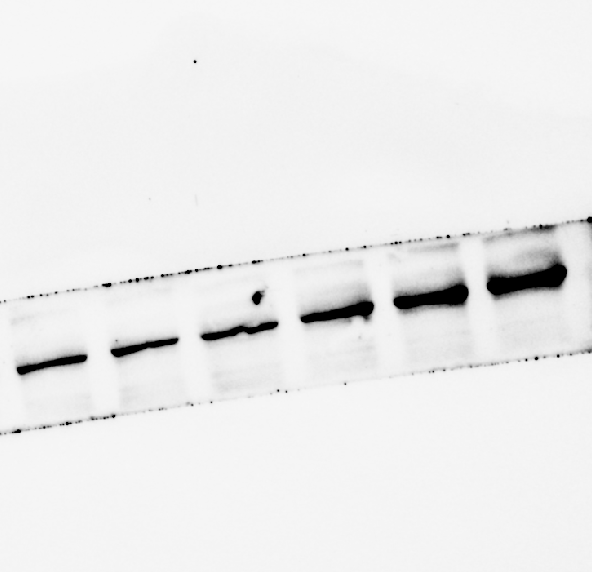

Supplement: Supplementary file 1 [file Data_Sheet_1.ZIP › raw data -/WB data/Fig. 2GPR78-I.tif]

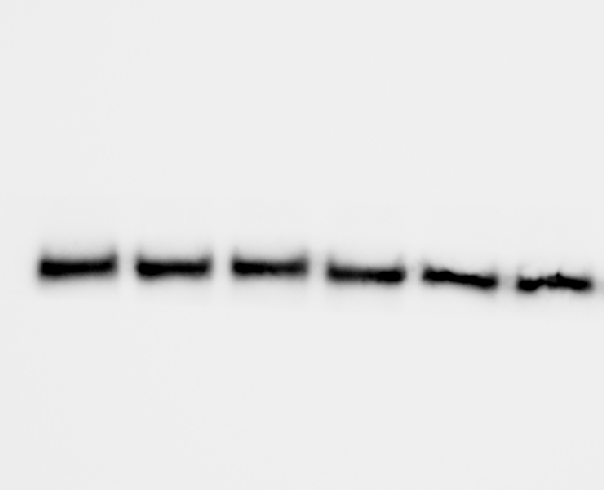

Supplement: Supplementary file 1 [file Data_Sheet_1.ZIP › raw data -/WB data/Fig. 2IRE1-I-1.tif]

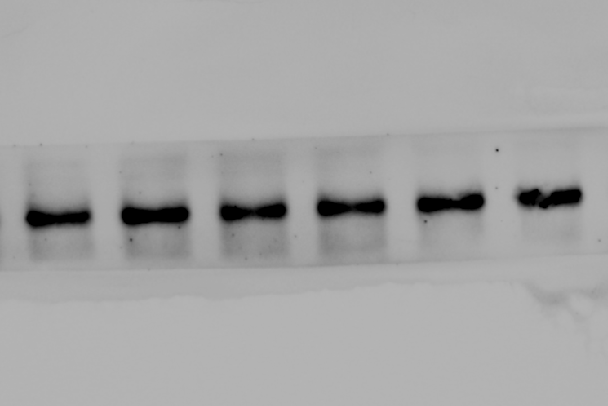

Supplement: Supplementary file 1 [file Data_Sheet_1.ZIP › raw data -/WB data/Fig. 2IRE1-I.tif]

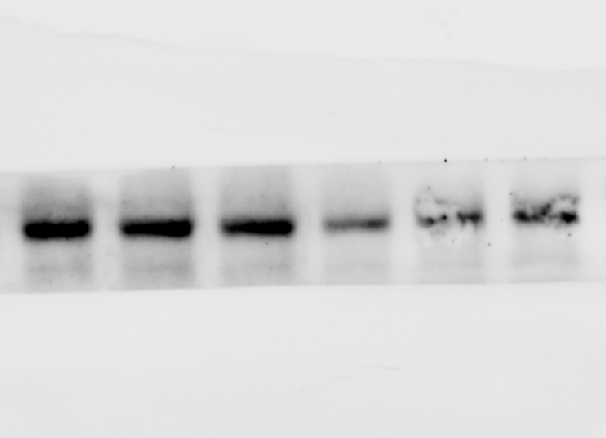

Supplement: Supplementary file 1 [file Data_Sheet_1.ZIP › raw data -/WB data/Fig. 2MUC2-GC.tif]

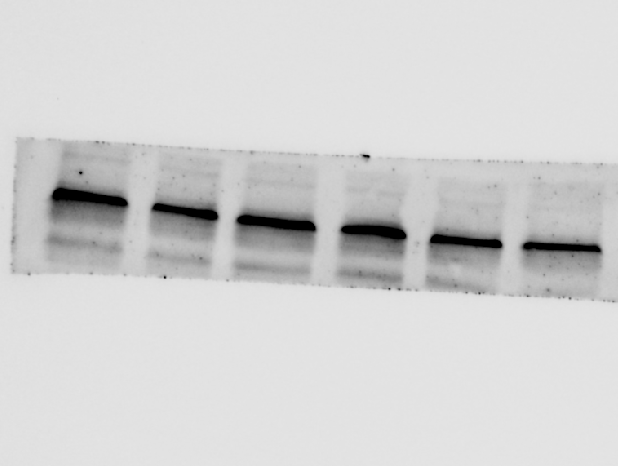

Supplement: Supplementary file 1 [file Data_Sheet_1.ZIP › raw data -/WB data/Fig. 2action-GC.tif]

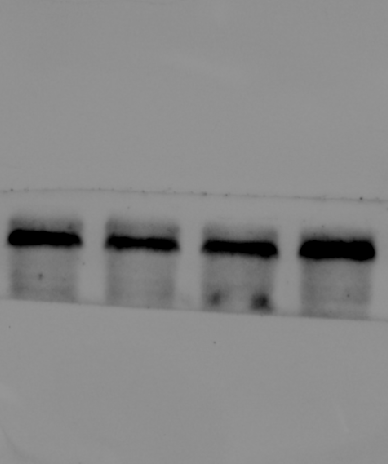

Supplement: Supplementary file 1 [file Data_Sheet_1.ZIP › raw data -/WB data/Fig. 5M-1.tif]

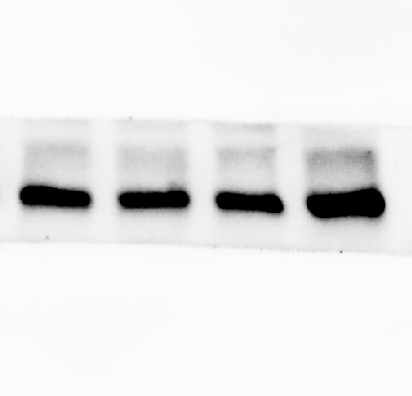

Supplement: Supplementary file 1 [file Data_Sheet_1.ZIP › raw data -/WB data/Fig. 5M-2.tif]

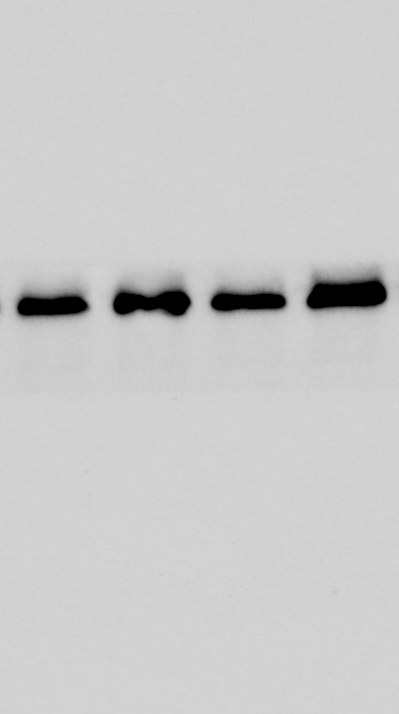

Supplement: Supplementary file 1 [file Data_Sheet_1.ZIP › raw data -/WB data/Fig. 5M-3.tif]

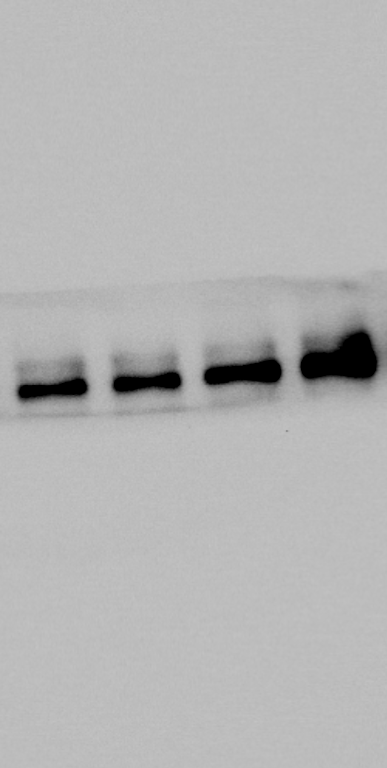

Supplement: Supplementary file 1 [file Data_Sheet_1.ZIP › raw data -/WB data/Fig. 5M-4.tif]

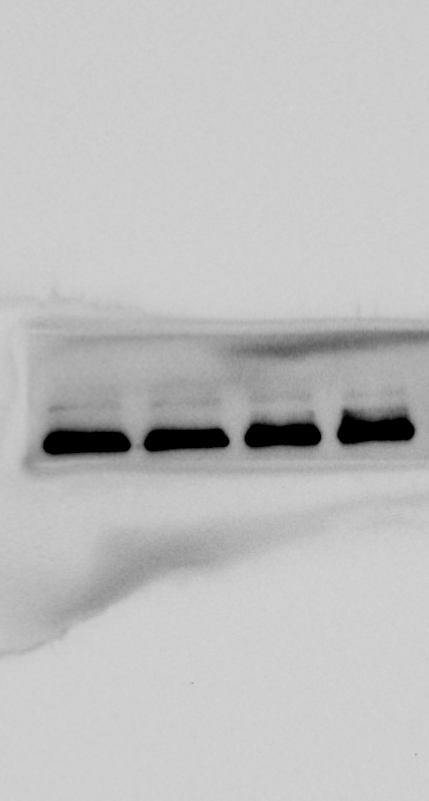

Supplement: Supplementary file 1 [file Data_Sheet_1.ZIP › raw data -/WB data/Fig. 5Maction.tif]

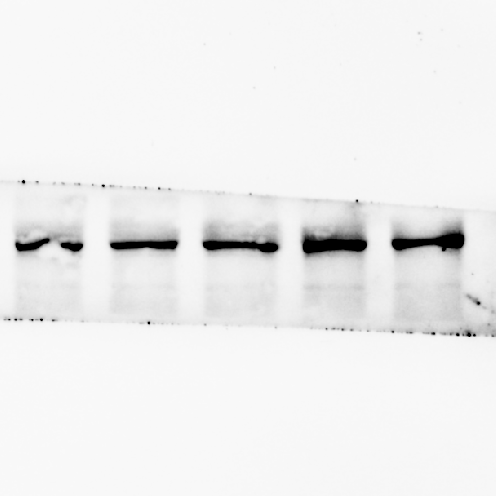

Supplement: Supplementary file 1 [file Data_Sheet_1.ZIP › raw data -/WB data/Fig. 6G-3.tif]

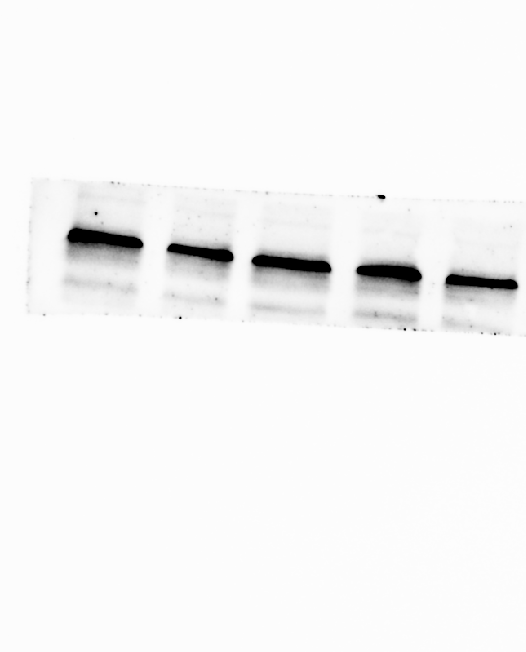

Supplement: Supplementary file 1 [file Data_Sheet_1.ZIP › raw data -/WB data/Fig. 6H-2.tif]

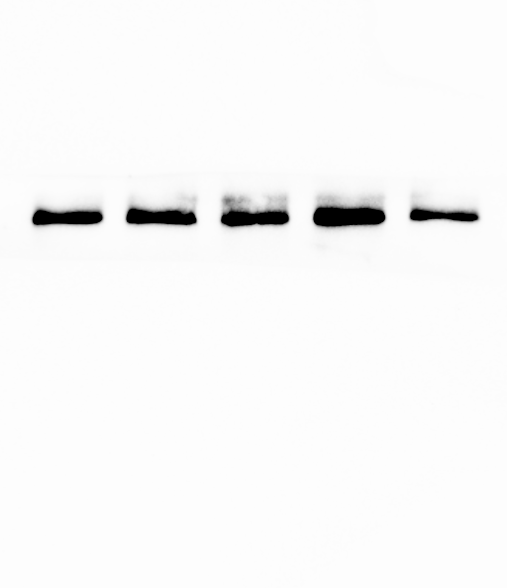

Supplement: Supplementary file 1 [file Data_Sheet_1.ZIP › raw data -/WB data/Fig. 6H-4.tif]

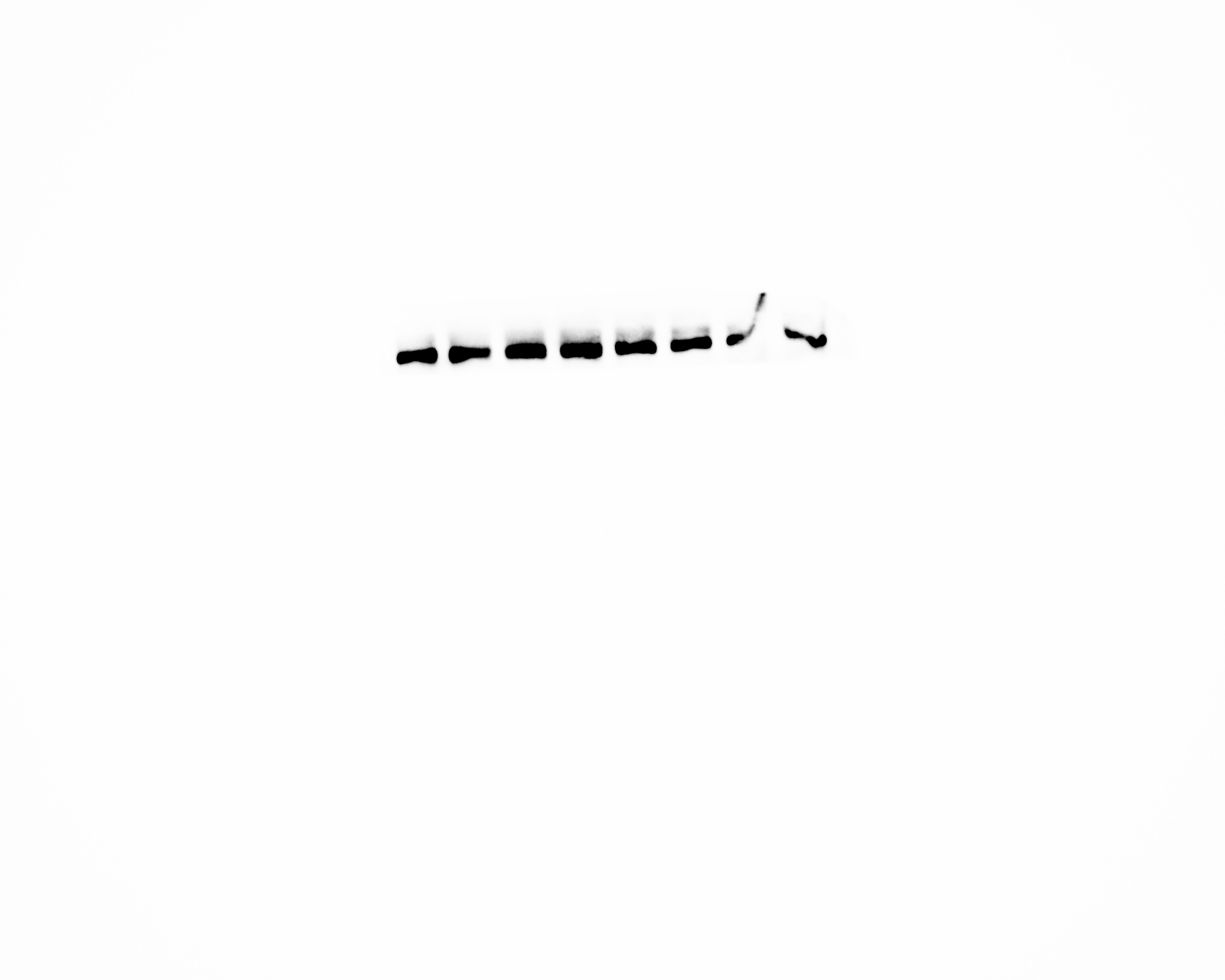

Supplement: Supplementary file 1 [file Data_Sheet_1.ZIP › raw data -/WB data/Fig. 6haction.tif]

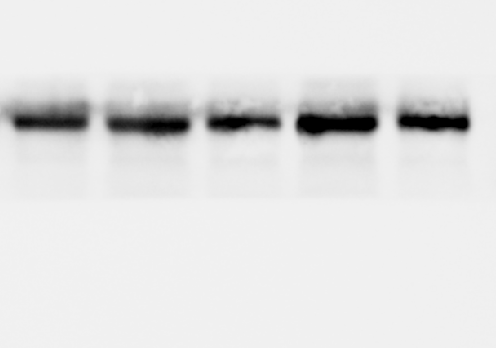

Supplement: Supplementary file 1 [file Data_Sheet_1.ZIP › raw data -/WB data/Fig.6H-1.tif]
